# Improving the Predictive Value of Phytochrome Photoequilibrium: Consideration of Spectral Distortion Within a Leaf

**DOI:** 10.3389/fpls.2021.596943

**Published:** 2021-05-24

**Authors:** Paul Kusuma, Bruce Bugbee

**Affiliations:** Crop Physiology Laboratory, Utah State University, Logan, UT, United States

**Keywords:** phytochrome, morphology, photobiology, far-red, photostationary state, phytochrome photoequilibrium

## Abstract

The ratio of active phytochrome (Pfr) to total phytochrome (Pr + Pfr), called phytochrome photo-equilibrium (PPE; also called phytochrome photostationary state, PSS) has been used to explain shade avoidance responses in both natural and controlled environments. PPE is commonly estimated using measurements of the spectral photon distribution (SPD) above the canopy and photoconversion coefficients. This approach has effectively predicted morphological responses when only red and far-red (FR) photon fluxes have varied, but controlled environment research often utilizes unique ratios of wavelengths so a more rigorous evaluation of the predictive ability of PPE on morphology is warranted. Estimations of PPE have rarely incorporated the optical effects of spectral distortion within a leaf caused by pigment absorbance and photon scattering. We studied stem elongation rate in the model plant cucumber under diverse spectral backgrounds over a range of one to 45% FR (total photon flux density, 400–750 nm, of 400 μmol m^–2^ s^–1^) and found that PPE was not predictive when blue and green varied. Preferential absorption of red and blue photons by chlorophyll results in an SPD that is relatively enriched in green and FR at the phytochrome molecule within a cell. This can be described by spectral distortion functions for specific layers of a leaf. Multiplying the photoconversion coefficients by these distortion functions yields photoconversion weighting factors that predict phytochrome conversion at the site of photon perception within leaf tissue. Incorporating spectral distortion improved the predictive value of PPE when phytochrome was assumed to be homogeneously distributed within the whole leaf. In a supporting study, the herbicide norflurazon was used to remove chlorophyll in seedlings. Using distortion functions unique to either green or white cotyledons, we came to the same conclusions as with whole plants in the longer-term study. Leaves of most species have similar spectral absorbance so this approach for predicting PPE should be broadly applicable. We provide a table of the photoconversion weighting factors. Our analysis indicates that the simple, intuitive ratio of FR (700–750 nm) to total photon flux (far-red fraction) is also a reliable predictor of morphological responses like stem length.

## Introduction

Light-emitting diodes (LEDs) provide a high degree of control over spectral output, which can be utilized to manipulate plant photoreceptors, but this manipulation requires an understanding of the photoreceptor activity. The action of phytochrome, the most well studied photoreceptor, has been extensively modeled (Sage, 1992), and our understanding continues to evolve (Sellaro et al., 2019; Smith and Fleck, 2019). In addition to predicting plant morphology in the field, phytochrome models must be able to predict morphology in controlled environments that can have unique background spectra.

Here we describe the historic and evolving modeling of phytochrome action that is largely based on stem/hypocotyl elongation. We discuss how these models have mostly ignored the issue spectral distortion by chlorophyll screening in green plants, and show that accounting for spectral distortion within leaves improves the predictive capability of classic phytochrome models.

### A Historic Review of Phytochrome Modeling

Models of phytochrome action were developed in parallel with its discovery. The first models included the photon absorbing pigment phytochrome and reaction partners (Borthwick et al., 1952), where two forms of phytochrome were interconverted by red (R) and far-red (FR) photons. Later, Hartmann (1966) provided a hypothesis to explain how phytochrome controlled the high irradiance response. He simultaneously irradiated hypocotyls with photons at two wavelengths, and explained the results with an estimate of the ratio of Pfr to Ptotal [called phytochrome photoequilibrium (PPE) or the photostationary state (PSS)], where Ptotal is the sum of Pr plus Pfr.

Hartmann’s work was praised by Smith (1973, 1975), who hypothesized that the PPE ratio explained phytochrome regulated responses in mature plants in the natural environment. Morgan and Smith (1976) provided evidence for this hypothesis by showing a direct linear relationship between PPE and the log of the stem extension rate. Morgan and Smith (1979) went on to show that this log linear relationship generally held for multiple species that evolved in a range of environments with the exception of understory plants that evolved in woodland areas, which had either a reduced or absent response. Child and Smith (1987) further built upon this hypothesis, showing that the rapid percentage increase in stem extension rate after applying FR was linearly correlated with PPE.

Smith and collaborators either measured PPE directly in etiolated tissue (Morgan and Smith, 1976; Smith, 1990) or estimated it with the R:FR ratio (Morgan and Smith, 1978, 1979). It is now common to predict PPE from the spectral photon distribution (SPD) above the canopy and photoconversion coefficients: σ_R_ for the conversion of Pr → Pfr, and σ_FR_ for the conversion of Pfr → Pr. These coefficients are essentially probability functions that predict the likelihood of photon absorbance at a given wavelength and subsequent conversion to the other form. The calculation to estimate PPE following this method is as follows:

(1) $$\text{PPE}=\,\frac{\text{Pfr}}{\text{Ptotal}}\,=\,\frac{\sum_{\lambda =300\,\mathrm{nm}}^{\lambda =800\,\mathrm{nm}}I_{\lambda }\,\sigma_{\text{R},\lambda }}{\sum_{\lambda =300\,\mathrm{nm}}^{\lambda =800\,\mathrm{nm}}I_{\lambda }\,\sigma_{\text{R},\lambda }+\,\sum_{\lambda =300\,\mathrm{nm}}^{\lambda =800\,\mathrm{nm}}I_{\lambda }\,\sigma_{\text{FR},\lambda }}$$

Where *I*_λ_ is the incident photon flux density at wavelength, λ. Photoconversion coefficients are calculated from *in vitro* measurements of the photochemical properties of phytochrome including: (1) absorbance spectra, (2) an estimation/calculation of PPE under actinic red photons, (3) the extinction coefficient of Pr at the peak in the red region (about 668 nm), and (4) quantum yields of Pr → Pfr and Pfr → Pr. Different photochemical properties are provided in at least ten publications (see Mancinelli, 1986, 1988, 1994; Lagarias et al., 1987). Thus it is possible to derive different photoconversion coefficients (Here, the term photoconversion coefficient refers to what has historically been called the photochemical/photoconversion cross-section, whereas the term photoconversion coefficient historically refers to the photochemical cross-section divided by the natural log of 10. Because coefficient is a more friendly term we use this term instead of cross-section).

These photoconversion coefficients, however, are primarily based on phytochrome-A (phyA) and not phyB. The phyB photoreceptor is the primary phytochrome photoreceptor responsible for sensing and responding to shade in the natural environment (Legris et al., 2019). Although phyA plays a larger role during de-etiolation (Mazzella and Casal, 2001), only monogenic mutants deficient in phyB (compared to monogenic mutants deficient in phyA, phyC, phyD, or phyE) appear elongated when grown in white light indicating the dominant role of phyB past the stage of de-etiolation (Whitelam et al., 1993; Aukerman et al., 1997; Devlin et al., 1998, 1999; Franklin et al., 2003; Franklin and Quail, 2010). Some limited evidence suggests that the photochemical properties of phyA and phyB may be similar (Ruddat et al., 1997; Eichenberg et al., 2000). If so, the fact that the photoconversion coefficients are derived primarily from phyA may not be a significant concern.

Estimates of PPE using photoconversion coefficients and the SPD above the leaf were used by Park and Runkle(2017, 2018, 2019) whose data shows a linear (as opposed to log linear) relationship between estimated PPE and stem length in several ornamental species. Overall, PPE estimates have resulted in a negative relationship with stem length. One limitation of most previous studies is that they are typically performed under a single background SPD, and treatments often only change the amount of FR and occasionally the amount of R. Thus, the full extent of the reliability of estimated PPE to predict morphology has not been determined.

### Recent PPE Modeling Efforts

#### The Three-State Model

The model described above (PPE = Pfr / Ptotal) is called *the two- state model*. A more complex model considers the dimerization of the phytochrome molecule in which the two arms of the dimer are activated independently. This is called *the three-state model*. It assumes only the Pfr–Pfr homodimer (called *D*_2_) is the active form, while the Pr–Pr homodimer (*D*_0_) and the Pr–Pfr heterodimer (*D*_1_) are both inactive. Therefore *the three-state model* at photoequilibrium is equal to *D*_2_/(*D*_0_+*D*_1_+*D*_2_), which can be calculated by squaring PPE calculated by *the two-state model* (PPE^2^; Mancinelli, 1988, 1994). Although there is sufficient evidence to suggest that phytochrome exists as a dimer (Jones and Quail, 1986; Brockmann et al., 1987; Rockwell et al., 2006) the evidence that *D_2* is the only active form is at present only based on mathematical analysis (Klose et al., 2015), and further investigation is required.

#### The Cellular Model

Thermal reversion, phytochrome destruction and nuclear body association/disassociation can either reduce or stabilize the pool of active phytochrome (Rausenberger et al., 2010; Klose et al., 2015). When these factors are considered the model is referred to as *the cellular model*. Sellaro et al. (2019) described that these other factors mainly play a role at low photon fluxes and/or high temperature, while only photoconversions apply at sufficiently high photon fluxes and low enough temperature. This model is thoroughly described in Smith and Fleck (2019). These complex models have yet to be used in applied research.

### Spectral Distortion Within Leaves

Leaves/cotyledons, and not stems/hypocotyls, were shown to be the primary site of red and far-red perception in *Cucumis sativus* (Black and Shuttleworth, 1974), *Sinapis alba* (Casal and Smith, 1988a), *Arabidopsis thaliana* (Tanaka et al., 2002; Endo et al., 2005), and *Brassica rapa* (Procko et al., 2014), while both organs were shown to be perceptive in a separate study in *Sinapis alba* (Casal and Smith, 1988b) and the epicotyl was shown to be the primary site of perception in *Vigna sinensis* (García-Martínez et al., 1987). Upon far-red perception in leaves/cotyledons, signals (including auxin) are transported to the stem/hypocotyl to induce elongation (Tanaka et al., 2002; Procko et al., 2014). From these data, it seems likely that phytochrome in the leaves/cotyledons play a dominant role in controlling stem elongation, with stems/hypocotyls playing a secondary role.

**FIGURE 1:**
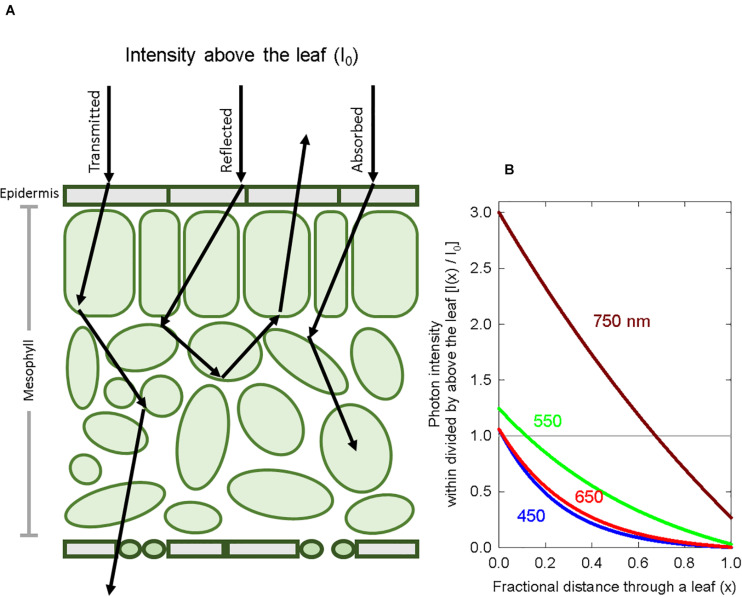
Basic principles of spectral distortion and photon scattering within a leaf. **(A)** A diagram of a cross section of a leaf showing the scattering of photons, which are eventually transmitted, reflected or absorbed. **(B)** A graphical representation of photon intensity at wavelengths 450, 550, 650, and 750 nm as a function of leaf depth. Because of reflection, diffraction and refraction, the photon intensity in the top layers of a leaf can exceed the intensity above the leaf. The Kubelka-Munk theory was used to calculate photon intensity with depth. The grey horizontal line represents the photon intensity just above the leaf.

A major issue with using photoconversion coefficients to estimate PPE is that they are applied to the SPD above the leaf, and not the SPD at the phytochrome molecule, which is distorted by chlorophyll and other pigments, as well as cell walls. Photons are scattered within leaves making the light diffuse within leaves (Figure 1). Due to this internal reflection, refraction and diffraction, leaves act as “light traps” wherein the photon intensity in the epidermis can exceed the intensity above the leaf by several fold (Seyfried and Fukshansky, 1983; Vogelmann, 1994). Because the term attenuation specifically refers to a decrease in the photon intensity, we use the term distortion to describe spectral changes in leaves.

Both Morgan and Smith (1978) and Gardner and Graceffo (1982) discuss chlorophyll screening issues stating that estimates of PPE (above the leaf) are only accurate for the top epidermal layer of cells within a leaf. Gardner and Graceffo (1982) suggested that the functional layer of phytochrome must be near the outer epidermal layer because of the linear relationship between PPE and the log stem extension rate seen in Morgan and Smith (1976). These assumptions are invalid because spectral distortions still occur in the epidermis, and additionally, several studies have shown that the peaks of phytochrome regulated action spectra shift to lower wavelengths than the peak absorbance of extracted phytochrome, indicating that some degree of spectral distortion occurs within leaves. For example, Kasperbauer et al. (1963) observed that inhibition of flowering in *Chenopodium rubrum* was most affected by night break lighting at 645 nm, instead of the expected 660 (or 668) nm, an effect they attributed to spectral filtering by chlorophyll. Similarly, Jose and Schäfer (1978) found that 630 nm photons induced the shortest hypocotyls and internodes in green tissue.

Several attempts have been made to account for spectral distortion within a leaf, especially via Kubelka-Munk theory. The Kubelka-Munk theory describes light propagation within a scattering medium like a leaf (Vogelmann, 1994). It simplifies to the Beer-Lambert law if extinction and scattering coefficients are assumed to be constant and not dependent on fractional distance through the leaf (Evans, 1995). Holmes and Fukshansky (1979) modeled PPE through a green leaf using the Kubelka-Munk theory and estimated that PPE decreased by about 40% as it moved through a leaf under full sunlight. Later, Kazarinova-Fukshansky et al. (1985) used the Kubelka-Munk theory to develop distortion functions that describe photon gradients within zucchini cotyledons. These distortion functions can be multiplied by the phytochrome photoconversion coefficients to develop weighting factors that can be used to predict the action spectra of phytochrome conversions within a certain layer of cotyledon tissue based on the incident photon flux above the leaf. Little has been done to test predictions of PPE with these weighting factors using experimental data. As such, despite the efforts of Kazarinova-Fukshansky et al. (1985), above-the-leaf estimates are still regularly employed.

Because the degree of spectral distortion depends on the specific layer of the leaf, it is important to ask whether all phytochrome is “functional”. The epidermis has been shown to control the rate of elongation (Kutschera and Niklas, 2007; Savaldi-Goldstein et al., 2007), but whether the epidermis is where the light signals are perceived, especially in leaf/cotyledon tissue, remains undetermined. Phytochrome is expressed in all tissue (Somers and Quail, 1995), but Kim et al. (2016) concluded that only phytochrome in epidermal tissue (of the hypocotyl) controlled elongation under continuous R light and end-of-day FR. This conclusion was based on transgenic lines of *Arabidopsis thaliana* that controlled *PHYB* expression using hypocotyl-tissue-specific promoters, effectively limiting phyB to specific layers of hypocotyl tissue (i.e., epidermis, cortex, endodermis, and vasculature). Endo et al. (2005) similarly expressed phytochrome in tissue specific organs and found that mesophyll-located phytochrome (in the cotyledons) controlled elongation.

Our objective was to use models of spectral distortion within a leaf (for both epidermal-located phytochrome and homogeneously distributed phytochrome) to improve the predictive relationship between PPE and morphological parameters.

## Materials and Methods

Two studies were conducted:

1. Cucumber plants were grown for 10–15 days in growth chambers with unique spectral backgrounds and different doses of FR (long-term study).
2. Elongation of photobleached and green cucumber seedlings were compared after 2 days in the growth chambers with a gradient of FR (short-term photobleaching study).

In both studies, multiple models of spectral distortion were used to predict PPE in specific layers of tissue.

### Plant Materials

#### Long-Term Study

Tomato, lettuce, spinach, soybean, and cucumber were screened for sensitivity to FR by applying either a low dose or no added FR. Cucumber was the most sensitive species to FR and was selected for further study (example data from one tomato study is shown in Supplementary Figure 1).

Seeds of cucumber (*Cucumis sativus* cv. Straight Eight) were planted into 1.7 L pots with a 1:1 mixture of peat/vermiculite by volume amended with 1.6 g per liter of dolomitic lime and 0.8 g per liter Gypsum (CaSO_4_). Cotyledons emerged 4 days after planting and pots were moved from the greenhouse to the growth chambers (Supplementary Figure 2).

#### Short-Term Photobleaching Study

Nine cucumber seeds were germinated on black felt saturated with nutrient solution (Utah hydroponic refill solution for dicots, USU Crop Physiology Laboratory, 2020) in each of 22 germination boxes (18 × 16.5 cm^2^) at 21°C. Black felt was used to minimize ground reflection so photons would primarily enter the cotyledons from above (Supplementary Figure 3). After 3 days the radicle had emerged, and nutrient solution was re-applied, with half of the germination boxes (11 boxes) receiving 50 μM norflurazon in the nutrient solution. Norflurazon is an herbicide that blocks the synthesis of carotenoids, leading to photobleaching in high light, eventually killing the plant. Seeds were then moved into pretreatment conditions: two norflurazon treated and two non-treated boxes were moved into the dark and the remainder of the boxes were moved into a growth chamber with a continuous photosynthetic photon flux density (PPFD) of about 500 μmol m^–2^ s^–1^ (Supplementary Figure 4) and a temperature of 20°C to finish emerging. 12% of the seeds either did not germinate or were not vigorous and all boxes had at least 6 seedlings. After 3 days in the pretreatment the norflurazon treated seedlings appeared white with an average hypocotyl length of 1.4 cm and the non-photobleached seedlings had an average hypocotyl length of 1.2 cm. 3 days in continuous light reduces the concentration of highly light-labile phyA, which was shown to be reduced by 50- to 100-fold after 12 h under low red photon flux and was below detectable limits after 7 days in white light (Sharrock and Clack, 2002). This ensured that responses were primarily caused by phyB. The germination boxes were placed in seedling trays with one photobleached and one non-photobleached germination box in each tray. Trays were then placed in the growth chambers for 48 h. This was repeated four times.

### Environmental Conditions

#### Long-Term Study

Temperature was maintained at 27/22°C day/night. Plants were watered as needed (typically every 2–3 days) with a complete nutrient solution at a concentration of 120 mg N per L (Peter’s professional 20-10-20, 20N-4.4P-16.6K; Allentown, PA). Potassium silicate (AgSil16H; Certis United States; Columbia, MD, United States) was added to the nutrient solution at 0.3 mM Si. Chambers were enriched to 850 ppm CO_2_. All studies contained six replicate plants per treatment. Plants were rotated every other day to minimize any position effects in the chamber. Individual plants were analyzed as replicates. Plant density was 20 plants per square meter.

#### Short-Term Photobleaching Study

Temperature was maintained at a constant 20°C. The norflurazon treated seedlings lost turgor at low humidity so water was added to the tray and the tray was covered to raise the humidity. Condensation formed on the lid of the seedling trays.

### Spectral Treatments

**FIGURE 2:**
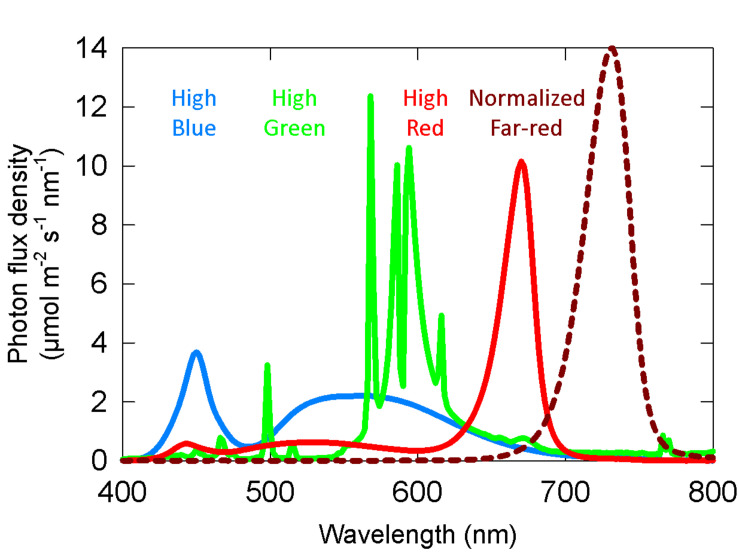
Representative spectral photon distributions (SPD) of the high blue, high green, and high red spectra used in both the long-term and short-term studies. The dashed dark red line is the SPD of the far-red, which was variable among studies.

Spectral measurements were made with a spectroradiometer (PS-300; Apogee instruments; Logan, UT, United States). For both the long-term and the short-term studies, three growth chambers (1.25 × 0.9 × 1.2 m^3^, L × W × H) provided separate background SPD from either cool white LED fixtures, 400-W high-pressure sodium (HPS) fixtures, or white + red LED fixtures. These background spectral distributions are common in controlled environment agriculture and are referred to here as high blue (cool white LED), high green (HPS) and high red (white + red LED). Spectral data for these background spectra are provided in Table 1 and Figure 2.

**TABLE 1:** Ratios of colors for the three spectral backgrounds.

Treatment	% BLUE	% GREEN	% RED	Total
	$\frac{\sum 400-499\,\text{nm}}{\sum 400-700\,\text{nm}}$	$\frac{\sum 500-599\,\text{nm}}{\sum 400-700\,\text{nm}}$	$\frac{\sum 600-699\,\text{nm}}{\sum 400-700\,\text{nm}}$	
HIGH BLUE	29	48	23	100
HIGH GREEN	6	52	42	100
HIGH RED	7	12	81	100

#### Long-Term Study

**FIGURE 3:**
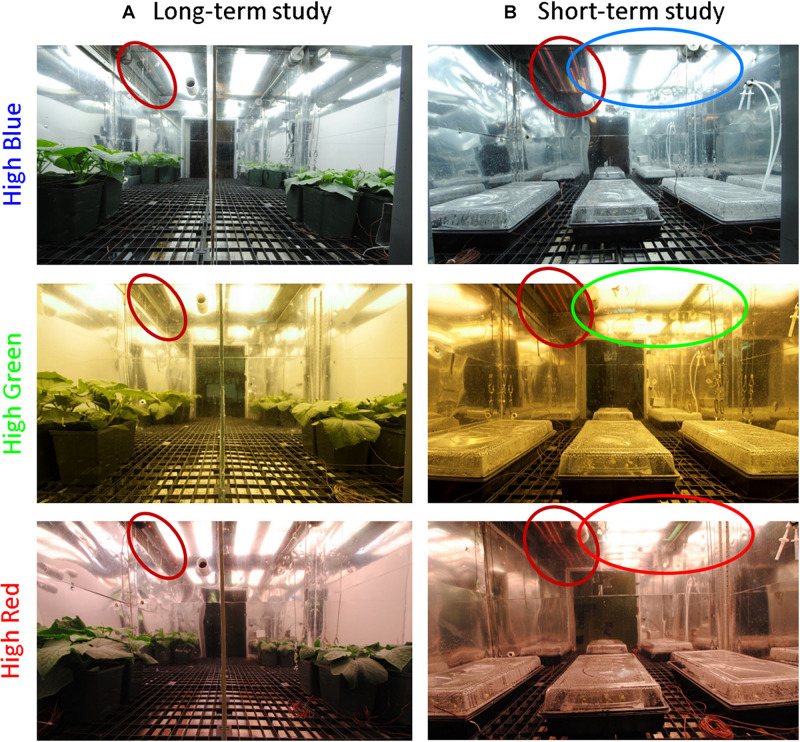
**(A)** Example photo of the plants in the high blue, green, and red chambers from the long-term studies. In this example there was no added far-red (FR) on the right side in each chamber. Treatments were randomized among studies. Each chamber was divided in half to supply two doses of FR. FR LEDs are circled in red. There is a second FR LED on the other side of the background LED (out of view, see Supplementary Figure 2) to improve the uniformity of FR. Additionally, in studies with higher fractions of FR, LEDs were placed across the top of the chamber. Uniformity within and between treatments was ensured by dimming lamps with either power supply capabilities or neutral density window screen. To achieve uniformity of SPD and extended photosynthetic photon flux density (ePPFD), the plants were grown on the sides of each half-chamber. **(B)** Photo of the experimental set-up for short-term photobleaching seedling study. Each chamber was provided with a high dose of FR on one side of the chamber and the background light source on the other side of the chamber. This provided a gradient of percent far-red decreasing from left to right. FR LEDs are circled in red, and the background light source is circled in its respective color. Seedlings (in germination boxes) were kept in seedling trays that were brought to a high relative humidity by placing water in the bottom of the tray.

Each chamber was separated in half with a white reflective board to provide a higher and lower level of FR from LEDs (peak of 730–735 nm). This allowed for two fractions of FR in each trial in time. Cucumber plants in the chambers at the end of one of these studies are shown in Figure 3A. The FR fraction was then varied among trials to achieve a collective range of one to 45% FR across seven replicate trials for a total of 14 doses of FR in each spectral background. Using regression analysis with plant rotation, this provided 84 replicates (six replicate plants × 14 doses of FR) for each spectral background.

Percent far-red (FR fraction) was calculated as:

(2) $$\mathrm{Percent}\,\mathrm{far}\,\mathrm{red}=\frac{\mathrm{FR}\,\mathrm{flux}\,\left(701-750\,\mathrm{nm}\right)}{\text{ePPFD}\,\left(400-750\,\mathrm{nm}\right)}\,\times 100$$

Because FR photons are photosynthetically active (Zhen and van Iersel, 2017; Zhen and Bugbee, 2020), the extended photosynthetic photon flux density (ePPFD: 400–750 nm) was kept constant among studies. This meant that as FR increased, the traditional PPFD (400–700 nm) decreased. For ePPFD, a cut-off wavelength of 750 nm may slightly overestimate photosynthetic photons (Zhen et al., 2018), but this definition is adequate for FR from LEDs. The average ePPFD was 400 and carefully adjusted so that it varied less than 10 μmol m^–2^ s^–1^ among the background spectra in each study. The photoperiod was 16 h. Detailed spectral information showing the 1–45% FR is provided in Supplementary Table 1 and Supplementary Figure 5.

#### Short-Term Photobleaching Study

The background light fixtures were placed at the top of one side of the chamber and FR LED bars were placed on the other side of the chamber to provide a gradient of FR that increased from right to left while the PPFD increased from left to right. Seedling trays were placed on the left, middle and right sides of the chamber to obtain about 18, 31, and 50% FR for each background spectrum. A photo of the experimental set-up is provided in Figure 3B. The average ePPFD in these studies was 300 μmol m^–2^ s^–1^ and varied less than 15 μmol m^–2^ s^–1^ among the background spectra. The SPDs for these studies are shown in Supplementary Figure 6. Light was applied continuously for the whole 48 h treatment period.

### Estimation of PPE

We calculated PPE (assuming *the two-state model*) following Eq. 1. We used the photoconversion coefficients derived from the photochemical properties in Lagarias et al. (1987). These are different than other commonly-used photoconversion coefficients (Kelly and Lagarias, 1985; Sager et al., 1988) on an absolute scale, but are similar when normalized to the Pr peak. The absolute magnitudes of the photoconversion coefficients are only important if other rates of phytochrome dynamics, like thermal reversions, are considered.

### Estimation of the Three-State Model

We did not account for the additional factors in *the cellular model* proposed by Rausenberger et al. (2010) and modified by Klose et al. (2015). Sellaro et al. (2019) reported that when the temperature is 25°C, the cellular model reaches 99% of *the three-state model* (assuming only photoconversions) at a PPFD of about 450 μmol m^–2^ s^–1^, and when the temperature is 20°C, the cellular model reaches 99% of *the three-state model* at a PPFD of about 350 μmol m^–2^ s^–1^. These conditions are close to the environmental conditions used in these experiments. Therefore, we used the simplified *three-state model* assuming the temperature effects on phytochrome reversion were negligible. As mentioned previously, *the three-state model* is simply calculated by squaring PPE calculated by Eq. 1 (Mancinelli, 1988).

### Modeling Spectral Distortion Within a Leaf

We use spectral distortion functions derived from Kazarinova-Fukshansky et al. (1985) to predict spectral distortion at the phytochrome molecule under the assumption that “functional” phytochrome is either (1) only located in the epidermis (top 1% of the leaf), or (2) homogeneously distributed within all layers of the leaf. All curves from Kazarinova-Fukshansky et al. (1985) were obtained using GetData Graph Digitizer^1^. Kazarinova-Fukshansky et al. (1985) modeled spectral distortion using the Kubelka-Munk theory within 7 days old *Cucurbita pepo* cv. “Senator” (zucchini), a species closely related to cucumber.

**FIGURE 4:**
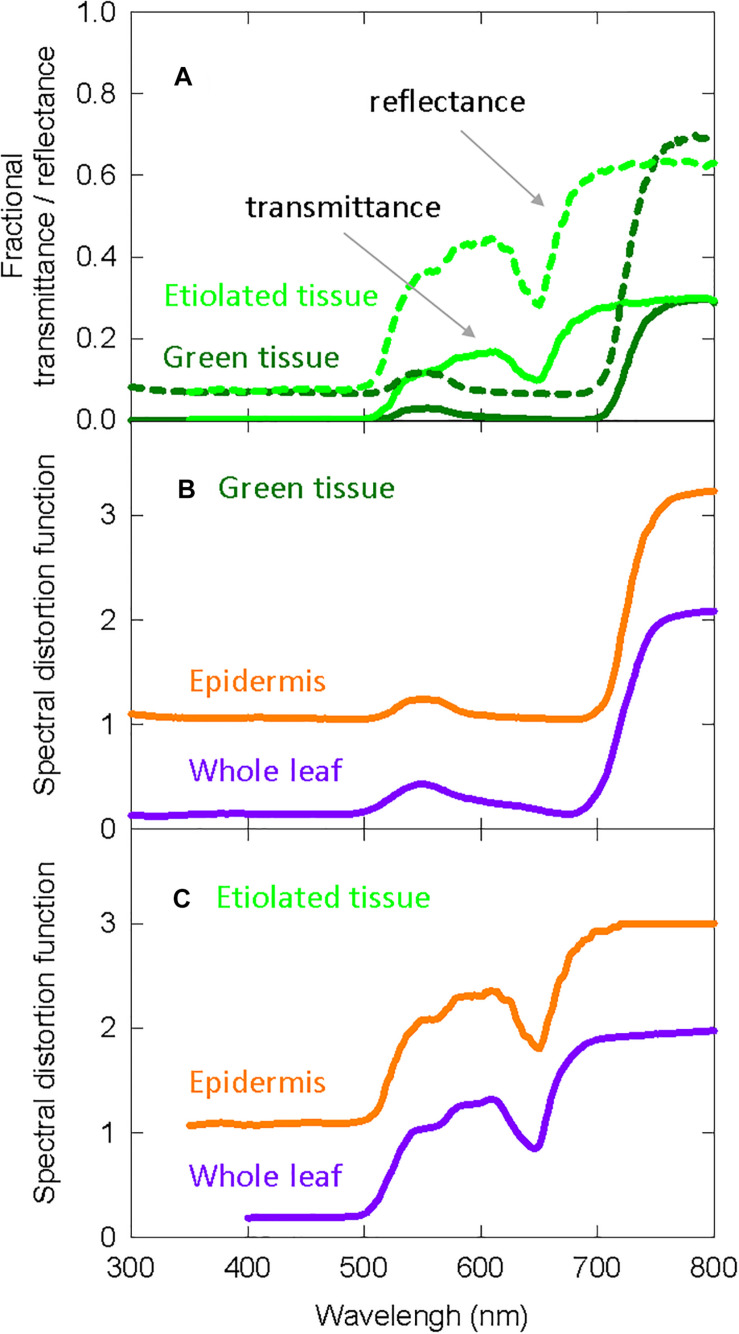
Spectral distortion functions developed from zucchini that were used in both the long-term and the short-term studies on elongation in cucumber. **(A)** Fractional transmittance (solid) and reflectance (dashed) spectra of green and etiolated zucchini cotyledons. Etiolated cotyledons represented norflurazon treated cotyledons. **(B)** Derived spectral distortion functions for green plants in epidermal tissue (orange) or the whole leaf (purple). **(C)** Derived spectral distortion functions in etiolated/white seedlings for epidermal tissue (orange) or the whole leaf (purple). All data are derived from Kazarinova-Fukshansky et al. (1985).

The Kubelka-Munk theory-based distortion functions use transmittance and reflectance measurements, so we include this data in Figure 4A for etiolated and green zucchini seedlings (Kazarinova-Fukshansky et al., 1985). Figure 4B shows the distortion functions for green plants assuming “functional” phytochrome is (1) only in the epidermal tissue (orange lines) or (2) homogenously distributed throughout the whole leaf (purple lines). Figure 4C shows the same distortion functions in etiolated tissue.

The photoconversion coefficients derived from Lagarias et al. (1987) were multiplied by the distortion functions to obtain modeled estimates of phytochrome conversion weighting factors (or action spectra) in specific layers of tissue (Eq. 3).

$$\mathrm{Photoconversion}\,\mathrm{weighting}\,\mathrm{factor}\,\mathrm{for}\,\mathrm{Pr}\,\left(\lambda \right)=\sigma_{\text{R}}\,\left(\lambda \right)\times \mathrm{Distortion}\,\mathrm{coefficient}\,\left(\lambda \right)$$

(3) $$\mathrm{Photoconversion}\,\mathrm{weighting}\,\mathrm{factor}\,\mathrm{for}\,\mathrm{Pfr}\,\left(\lambda \right)=\sigma_{\text{FR}}\,\left(\lambda \right)\times \mathrm{Distortion}\,\mathrm{coefficient}\,\left(\lambda \right)$$

### Plant Measurements

#### Long-Term Study

Plants were harvested when the stem length in the highest FR treatment was 25–30 cm long; this occurred 10–15 days after emergence. Stem length, petiole length and leaf area were recorded. Leaf area was measured with a leaf area meter (model Li-3000, LI-COR, Lincoln NE). Leaves, cotyledons and stems were separated and dried at 80°C for 2 days, after which dry mass was measured and percent leaf and percent stem dry mass were calculated by dividing the respective dry mass by the total dry mass.

Stems typically elongate following a sigmoidal curve (Fisher et al., 1996; Björkman, 1999) with exponential elongation in young plants (Morgan and Smith, 1978), followed by linear elongation, and finally, exponential rise to a maximum. This means that elongation is best described as a natural log function in the early stages of growth. For this reason early studies regularly used log-linear stem elongation rates to predict elongation as a function of PPE (Morgan and Smith, 1976, 1978, 1979). Thus in young plants, stem length at day (*t*) would be equal to:

(4) $$\mathrm{Stem}\,\mathrm{length}\,\left(t\right)=\mathrm{Stem}\,\mathrm{length}\,\left(i\right)\,e^{k\,t}$$

Where Stem length (*i*) is the initial length. We can then calculate the exponential extension coefficient (the natural log of the stem extension rate; lnSER or *k* in Eq. 4), assuming the initial stem length was equal to one, as follows:

(5) $$\text{lnSER}=\frac{\ln\left(\mathrm{Stem}\,\mathrm{length}\,\mathrm{at}\,\mathrm{harvest}\right)}{\mathrm{days}\,\mathrm{to}\,\mathrm{harvest}}$$

This equation was also used to calculate the leaf expansion coefficient (natural log of the leaf expansion rate; lnLER) and the petiole extension coefficient (natural log of the petiole extension rate; lnPER). Chlorophyll concentration was measured with a chlorophyll meter (model MC-100, Apogee Instruments, Logan, UT, United States).

#### Short-Term Photobleaching Study

Cucumber hypocotyl lengths were measured with a ruler to the nearest 0.5 mm before and after they were moved into the treatments. The change in hypocotyl length over 48 h was normalized to the elongation of the respective dark control:

(6) $$\mathrm{Elongation}\,\mathrm{relative}\,\mathrm{to}\,\mathrm{the}\,\mathrm{control}=\frac{L_{f}-L_{i}}{L_{c,t\,2}-L_{c,t\,1}}$$

Where *L*_*f*_ is the final hypocotyl length, *L*_i_ is the initial hypocotyl length, and *L*_*c,t1*_ and *L*_*c,t2*_ are the average hypocotyl lengths of the dark controls before or after the cucumber seedlings were placed in the treatments. The change in hypocotyl length was normalized to its respective control (with or without applied norflurazon) due to the finding of Casal (1995) in which norflurazon treated seedlings grown in the dark were 15–20% shorter than untreated seedlings. For each replicate in time, the elongation relative to the control for all the seedlings in each treatment were averaged together.

### Statistics

All data was analyzed using R statistical software (R Foundation for Statistical Computing; Vienna, Austria). Correlations were determined by calculating the *r*^2^ value of a trend-line through the data. Trend-lines used either linear or exponential decay functions. Data was analyzed using a mixed effect linear model using *lmer* and *Anova* functions with the F statistic judged to be significant at *p* < 0.05. The background spectra (e.g., high blue) were treated as a categorical variable, while different methods for analyzing the effect of FR were treated as a continuous variable. Two examples of methods for analyzing the effects of far-red include percent FR and PPE modeled above the leaf. Chambers and replicates were treated as random factors.

## Results

### Long-Term Study

**FIGURE 5:**
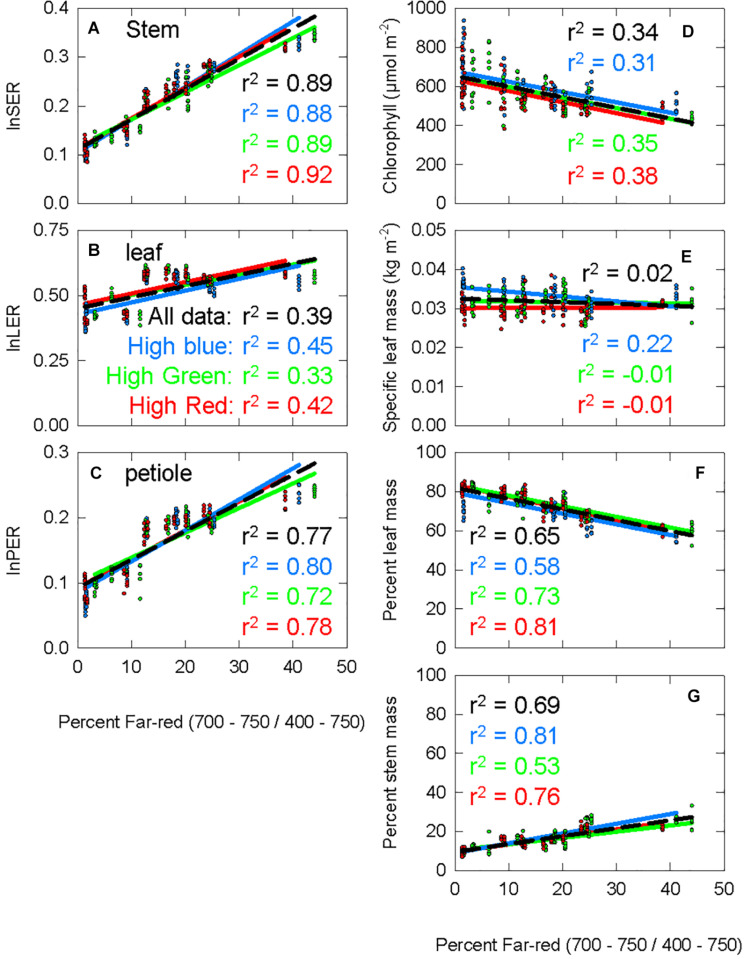
The response of seven physiological parameters to increasing percent far-red. The blue data and lines come from the chamber with spectral background containing a high portion of blue photons (high blue), the green data comes from the high green chamber, and the red data comes from the high red chamber. The *r*^2^ value for each background is shown with the respective color. The black dashed line is a trend line running through all the data from all three background light sources, with the corresponding *r*^2^ shown in black. **(A)** The stem extension rate constant (the natural log of the stem extension rate, log of the stem extension rate (lnSER); described in Eqs 4, 5). **(B)** The leaf expansion rate constant, calculated following the same method as lnSER, but using leaf area at harvest instead of stem length. **(C)** Petiole extension rate constant, calculated following the same method as lnSER, but using petiole length at harvest instead of stem length. **(D)** Chlorophyll concentration at harvest. **(E)** Specific leaf mass, leaf mass divided by leaf area. **(F)** Percent leaf mass, leaf mass divided by total shoot mass **(G)** percent stem mass, stem mass divided by total shoot mass.

The percent far-red ranged from less than 2% (which is only obtainable under LEDs) to 45% (typical of canopy shade). Figure 5 shows the response of seven morphological parameters to increasing percent FR under three diverse spectral backgrounds. lnSER, lnLER, lnPER, and percent stem mass all increased with increasing percent FR (Figures 5A–C,G). Chlorophyll concentration and percent leaf mass both decreased with increasing percent FR (Figures 5D,F). Specific leaf mass, which is calculated by dividing leaf mass by leaf area and is an indicator of leaf thickness, was unaffected by percent FR (*p* = 0.19, Figure 5E). Because lnSER had the highest correlation with percent FR, it was used as the response variable for models of PPE within leaf tissue.

#### Accounting for Spectral Distortions in Predictions of PPE

**FIGURE 6:**
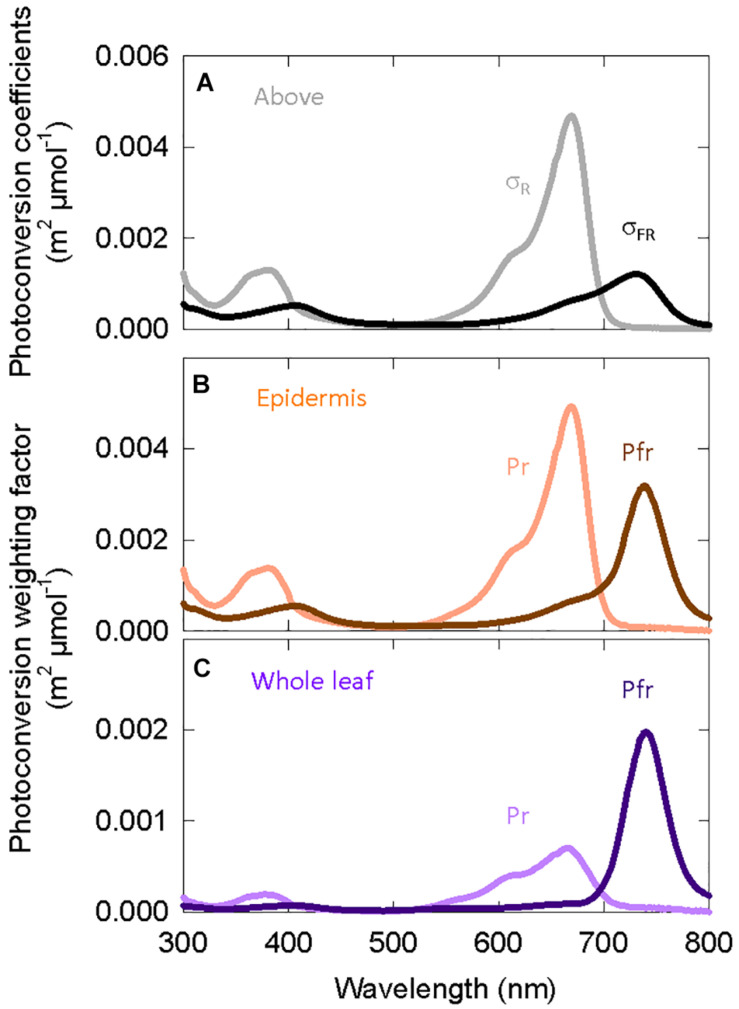
**(A)** Photoconversion coefficients derived from Lagarias et al. (1987). These are used to estimate PPE above the canopy. The other two graphs are the photoconversion weighting factors for phytochrome that is **(B)** only in epidermal tissue or **(C)** homogeneously distributed through all leaf/cotyledon tissue.

Multiplying the spectral distortion functions (Figure 4B) by the photoconversion coefficients (Eq. 3) provides weighting factors that predict local phytochrome conversions within a specific layer of tissue for a given SPD above the leaf (Figure 6). It is important to note that (a) the photochemical properties of phytochrome, and thus the photoconversion coefficients, have not changed and that (b) if no spectral distortion occurs within a leaf, then the photoconversion weighting factors are equal to the photoconversion coefficients.

Figure 6 shows that weighting factors for Pfr (Pfr → Pr) increase relative to Pr (Pr → Pfr) as the location of phytochrome moves from the epidermis to all leaf/cotyledon tissue. The weighting factors for Pr do not significantly shift the peak of action away from about 668 nm.

**FIGURE 7:**
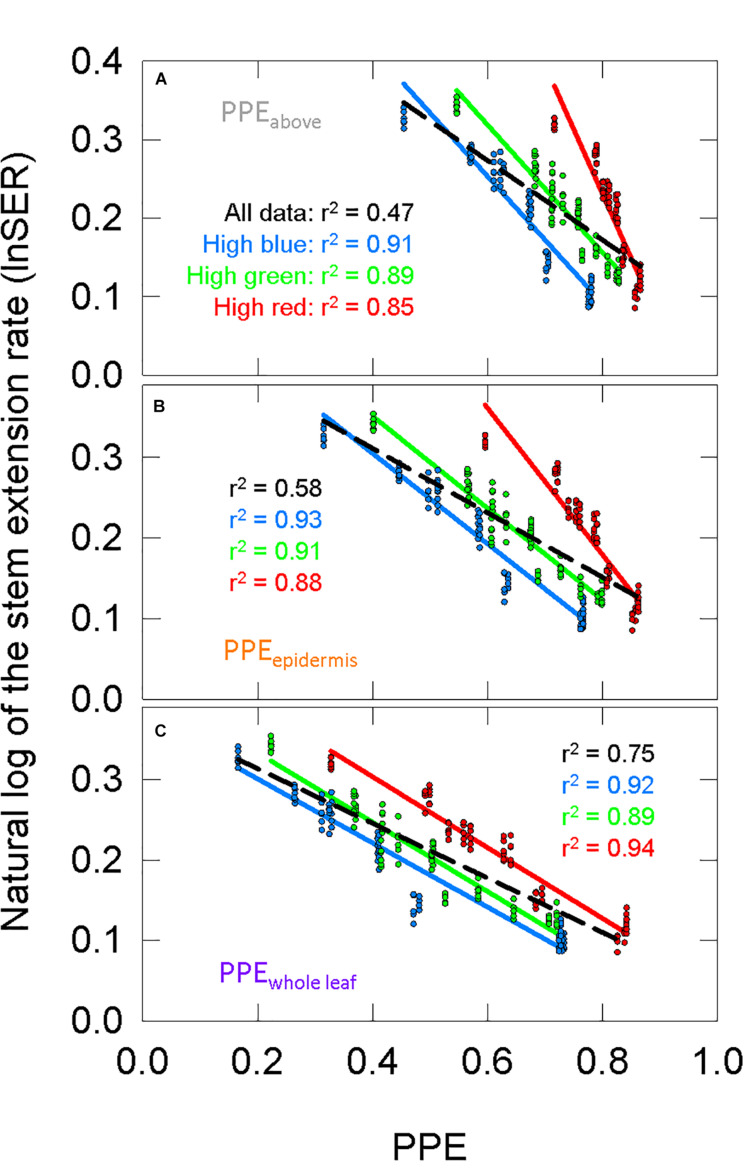
The response of the natural lnSER to changes in the estimate of phytochrome photoequilibrium (PPE) in multiple layers of tissue. PPE is calculated with *the two-state model*. See Figure 5 for an explanation of colors. **(A)** The relationship between PPE_above_ and lnSER. This is the most common method to model phytochrome activity using the spectral photon distribution above the leaf. Panels **(B,C)** use estimates of PPE for phytochrome that is **(B)** in epidermal tissue or **(C)** homogeneously distributed through the whole leaf.

Using σ_R_ and σ_FR_ (Figure 6A) in Eq. 1 or substituting them with the Pr and Pfr weighing factors (Figures 6B,C) produces estimates PPE in three layers: PPE_above_, PPE_epidermis_, and PPE_whole leaf_. We fit the lnSER data in Figure 5A to the estimates of PPE in these three layers assuming the commonly used *two-state model* (Figure 7). There was a high correlation between PPE estimated above the leaf (PPE_above_) and lnSER for any single background SPD (Figure 7A; *r*^2^ = 0.91, 0.89, and 0.85 for high blue, high green and high red, respectively). This relationship declines if PPE is compared to all the data (all three background spectra, dashed line, *r*^2^ = 0.47). The correlation between PPE and lnSER for any single background spectrum remained relatively unchanged when PPE was estimated in the epidermal leaf tissue (PPE_epidermis_) or the whole leaf (PPE_whole leaf_), but the relationship with all the data was improved when predicted within the leaf (Figures 7B,C). PPE_whole leaf_ produced the highest correlation between PPE and lnSER of all the data (*r*^2^ = 0.75, Figure 7C).

#### Comparison Between the Two-State and Three-State Models

**FIGURE 8:**
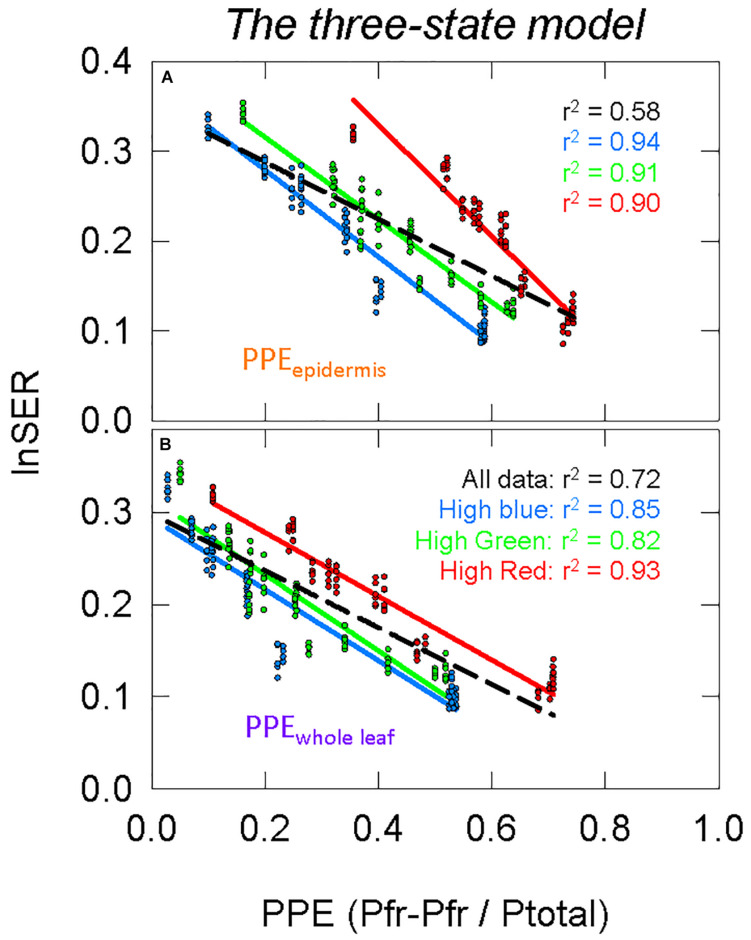
Modeling phytochrome activity with *the three-state model*. For this analysis, only phytochrome that is **(A)** in the epidermis or **(B)** homogeneously distributed within the whole leaf were considered. See Figure 5 for an explanation of colors.

*The two-state* and *three-state models* of phytochrome were compared assuming the active phytochrome was (a) in the epidermis and (b) homogeneously distributed in all the leaf tissue (Figure 8 compared to Figures 7B,C). Using regression analysis through all three spectral backgrounds, *the three-state model* did not improve the predictive power over the commonly used *two-state model* for any of the three assumed locations of phytochrome (*r*^2^ = 0.58 and 0.72 for PPE_epidermis_ and PPE_whole leaf_, respectively).

To further investigate differences between these four estimates of PPE (Figures 7B,C, 8), the slopes and offsets for the three individual background spectra (blue, green, and red lines) were compared using a linear mixed effects model, with the estimates of PPE as a continuous variable and the background spectrum as a categorical variable. There was a significant effect of the background spectrum on the prediction of lnSER for all four estimates of PPE, indicating that the offsets for the linear models were significantly different (*p* < 0.0001 in all cases). In the linear mixed effects model, an interaction effect between PPE and the background spectrum indicates that the slopes of the three lines are significantly different. This was the case for every model with the exception of only PPE_whole leaf_ using *the three-state model* (*p* = 0.25 compared to *p* = 0.033 for PPE_whole leaf_ using the *two-state model*, and *p* < 0.0001 for PPE_epidermis_ using both the *two* and *three-state models*). This means that the three lines (blue, green, and red) in this model of PPE (PPE_whole leaf_ using *the three-state model*) are not significantly different (nearly parallel).

### Short-Term Photobleaching Study

To further investigate the role spectral distortion by chlorophyll on estimates of PPE and subsequent stem or hypocotyl elongation, seedlings were grown with or without chlorophyll using the herbicide norflurazon.

**FIGURE 9:**
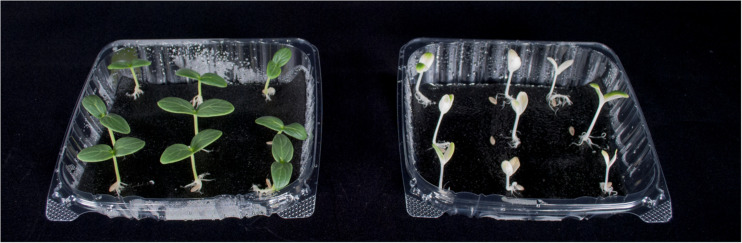
Representative plants at harvest from the short-term photobleaching study. Green seedlings are shown on the left and norflurazon-treated photobleached seedlings are shown on the right. There was some chlorophyll at the tips of some of the photobleached seedlings.

**FIGURE 10:**
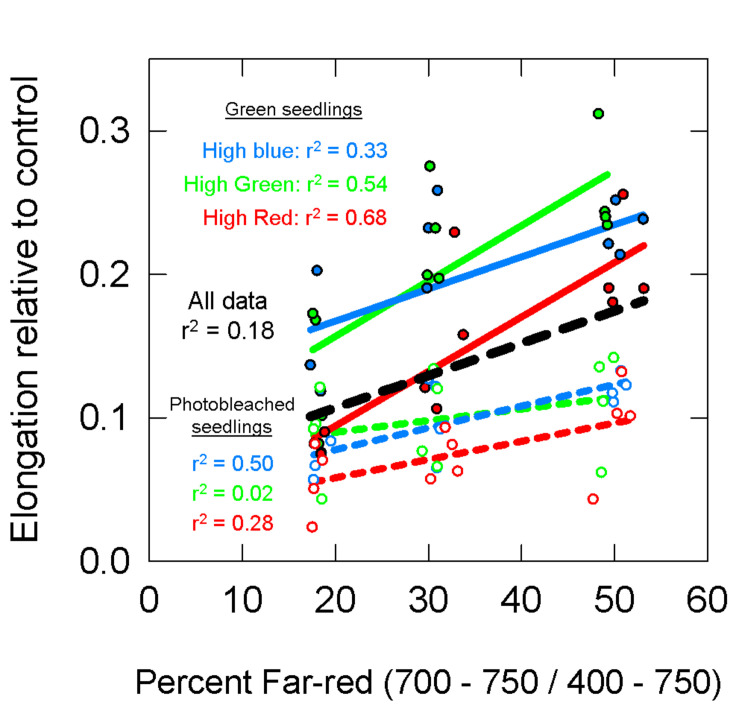
Elongation of green (closed circles, solid lines) and photobleached (open circles, dashed lines) seedlings over a 48 h period relative to dark controls. See Figure 5 for an explanation of colors. Data show that photobleached seedlings elongated less then green seedlings.

The photobleaching of the norflurazon treated seedlings was visually apparent, although some seedlings had chlorophyll at the tips of the cotyledons (Figure 9). Over the 48 h treatment period, the dark-grown norflurazon treated seedlings elongated an average of 8.5 cm, while the non-treated seedlings elongated an average of 9 cm. Elongation of seedlings in the light treatments relative to the dark controls are plotted as a function of percent FR in Figure 10. The photobleached seedlings elongated significantly less than the green seedlings, but a higher fraction of FR induced more elongation in both green and photobleached seedlings.

Spectral distortion functions for etiolated seedlings (Figure 4C) were used to calculate weighting factors for phytochrome conversions in either the epidermis or the whole leaf (Supplementary Figure 7). The photoconversion coefficients (Figure 6A) were substituted with the weighting factors for specific locations in green or etiolated cotyledons (Figures 6B,C and Supplementary Figure 7) in Eq. 1 to estimate PPE in these treatments.

**FIGURE 11:**
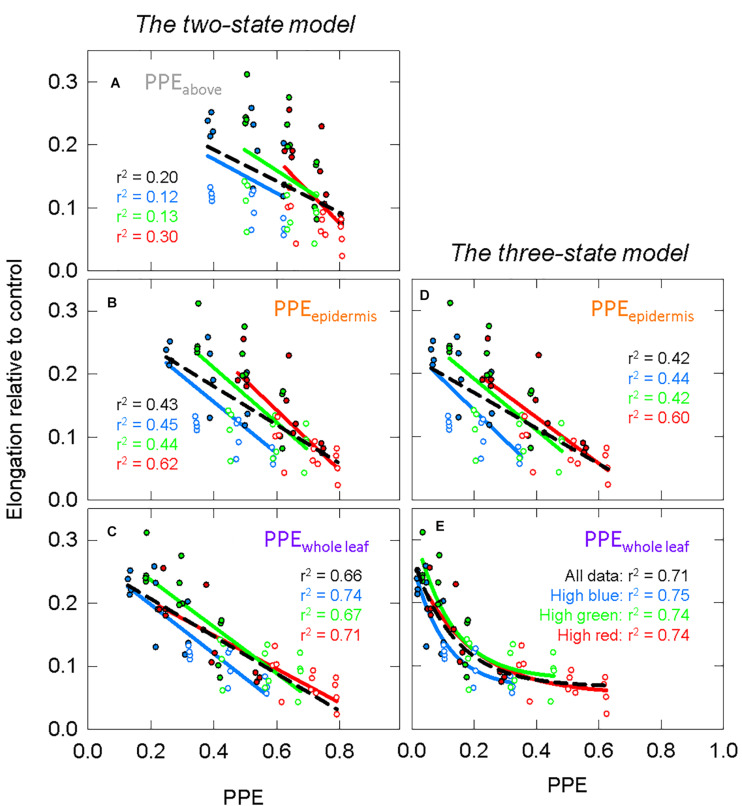
The response of green (closed circles) and photobleached (open circles) seedlings to models of PPE in specific leaf layers, using the same models as Figures 7, 8. See Figure 5 for an explanation of colors. Estimates of PPE are calculated using green or etiolated weighting factors. The green and photobleached seedlings from a single spectral treatment were combined for analysis. Panels **(A)** through **(C)** use estimates of PPE based on *the two-state model* for phytochrome that is **(A)** above the leaf, **(B)** in epidermal tissue or **(C)** homogeneously distributed in the whole cotyledon. Panels **(D,E)** use estimates of PPE based on *the three-state model* for phytochrome that is **(D)** in epidermal tissue or **(E)** homogeneously distributed in the whole cotyledons. All models use linear regression with the exception of **(E)**, which fits the date with exponential decay functions. Each point represents an average of 6–9 seedlings. There were four replications in time.

Figure 11 models the data in Figure 10 with these estimates of PPE. For this analysis, both the green and photobleached seedlings grown under a single spectral background (e.g., high blue) were combined together for regression analysis. Similar to the long-term study, PPE estimated above the cotyledon produced a poor correlation when run through all the data from all three spectral backgrounds (*r*^2^ = 0.20; Figure 11A), but unlike the long-term study, the regression through the data for a single spectral background also produced a poor correlation (*r*^2^ = 0.12, 0.13, and 0.30 for high blue, high green and high red, respectively). Compared to PPE estimated above the cotyledon (PPE_above_), the estimate of PPE within the epidermal tissue (PPE_epidermis_) provided a slight improvement in predictive ability (Figure 11B). Corroborating the results of the long-term study, the assumptions that “functional” phytochrome is homogeneously distributed within the whole leaf (PPE_whole leaf_) provided the best correlations between PPE and elongation relative to the dark controls (Figure 11C). This was true for both correlations using all the data and correlations using each individual background spectrum.

Similar to the comparison between *the three-state* and *the two-state models* in the long-term study, there was little difference between the correlation between PPE and elongation relative to the control in a specific layer of tissue using either model. *The three-state model* for homogenously distributed “functional” phytochrome required non-linear models to fit the data, and this resulted in a strong relationship (Figure 11E).

## Discussion

### Effects of Spectral Distortion on the Action Spectrum of Phytochrome Conversion

Kazarinova-Fukshansky et al. (1985) previously estimated the weighting factors for *in vivo* (either green or etiolated tissue) phytochrome photoconversions based on *in vitro* determinations of the photoconversion coefficients and their spectral distortion functions. They used the original photoconversion coefficients from Butler et al. (1964), which are based on partially degraded 60 kDa phytochrome rather than *native* 124 kDa phytochrome (Mancinelli, 1986). Therefore, the weighting factors from Kazarinova-Fukshansky et al. (1985) required updating using the most accurate photoconversion coefficients. Here, photoconversion coefficients calculated from the photochemical properties in Lagarias et al. (1987) were used.

The application of photoconversion weighting factors did not significantly shift of the Pr peak away from 668 nm. Therefore, we could not explain why Kasperbauer et al. (1963) or Jose and Schäfer (1978) observed shifts to 645 and 630 nm, respectively.

### Analysis of Phytochrome Models

The high correlations between PPE_above_ and lnSER for each individual background in the long-term study is similar to previous reports that kept the background spectrum constant, and only adjusted levels of R or FR (Morgan and Smith, 1976, 1978, 1979; Park and Runkle, 2017, 2018), but there is a low correlation when using PPE_above_ to broadly estimate lnSER under any spectral background (Figure 7A). By comparison, the convergence of lnSER data in Figures 7B,C indicate that models that account for spectral distortion within a leaf better predict phytochrome mediated plant responses under a broader range of spectral backgrounds.

Morgan and Smith (1978) found a linear relationship between PPE and lnSER when PPE was estimated under a leaf with a low chlorophyll concentration (380 μmol m^–2^), but they reported a departure from linearity at a high chlorophyll concentration (660 μmol m^–2^). Here, chlorophyll concentration in the leaves averaged 574 μmol m^–2^ across all treatments, although it ranged from 383 to 937 μmol m^–2^ and decreased as percent FR increased (Figure 5D). Using only the transmitted spectrum, the relationship between PPE and lnSER was non-linear (Supplementary Figure 8). Phytochrome in the upper layers of a leaf would have a lower “effective” chlorophyll concentration, and may be thought of as similar to the low chlorophyll leaf in Morgan and Smith (1978). Thus, the linear relationship between PPE and lnSER in the upper layers of leaf tissue (PPE_epidermis_) is similar to previous findings (Figure 7B).

Results from our short-term photobleaching study were similar to Holmes and Wagner (1981), who measured the percent inhibition of elongation (relative to dark controls) of green and noflurazon-treated *Chenopodium rubrum* seedlings grown under a single spectral background with added R or FR. As PPE increased from 0.3 to 0.8 in their study, inhibition of hypocotyl elongation increased (i.e., shorter hypocotyls) for both the treated and untreated seedlings, although the effect appeared reduced in the green seedlings. Additionally, when white light was applied along with R and FR, the green seedlings were taller than the norflurazon treated seedlings. Broadly, their results are similar to ours (Figures 10, 11A).

It is difficult to determine whether the relationship between PPE and lnSER should be linear (e.g., Figure 7C) or non-linear (e.g., Figure 11E). Activated phyB (Pfr) is translocated from the cytosol to the nucleus, where it interacts with numerous transcription factors including phytochrome interacting factors (PIFs), often inactivating or phosphorylating them (Legris et al., 2019). PIFs transcriptionally promote the expression of genes related to auxins, gibberellins, and cell walls, effectively leading to increased cell expansion (de Lucas and Prat, 2014). Thus, the down regulation of PIFs caused by higher relative concentrations of phyB–Pfr (high PPE) will cause a decrease in stem elongation, but with so many contributing factors, the exact relationship is difficult to determine. Additionally, post-transcriptional and translation regulation by phytochrome (Legris et al., 2019), the circadian control of phyB protein accumulation (Sharrock and Clack, 2002), and cytoplasmic roles of phytochrome (Hughes, 2013) all further complicate this relationship.

The assumption that “functional” phytochrome was homogeneously distributed throughout all leaf layers (whole leaf) provided better correlations with elongation than the assumption that “functional” phytochrome was only in the epidermis (Figures 7, 11). This corroborates the findings of Endo et al. (2005) who found that phyB expression in the mesophyll of the cotyledons restored the wild-type morphology in a *phyB* mutant. Kim et al. (2016) concluded that only phytochrome in the epidermis (of the hypocotyl) contributes to the control of hypocotyl elongation, but their results show a potential role for both epidermal and cortex located phytochrome in the control of hypocotyl elongation. Cortex and mesophyll cells are both “ground” tissue, comprising the majority of plant biomass. It seems likely that phytochrome in these cells (and the epidermis) modulate development in response to light signals, while phytochrome in vascular tissue does not (Endo et al., 2005; Kim et al., 2016).

The data presented here indicate that PPE estimated above a leaf is an inappropriate method for predicting phytochrome action. Under electric lights, above-the-leaf estimates of PPE are often above 0.8, which is higher than in sunlight. Some authors have concluded that the biological responses to treatments with PPE_above_ ranging from about 0.8 to 0.88 were likely not caused by phytochrome because it did not vary to a large degree (Barnes and Bugbee, 1992; Dougher and Bugbee,2001a,b; Cope and Bugbee, 2013). The proposed method of modifying the SPD that reaches phytochrome molecules demonstrates a high attenuation of R photons, resulting in lower rates of Pr → Pfr conversion than expected by above-the-leaf estimates. A re-evaluation of previous studies may be warranted.

### Consideration of More Recent Models: Three-State and Cellular Models

In the studies reported here, the intensity was kept close to the threshold intensities for a given temperature described by Sellaro et al. (2019) in order to minimize the contribution of thermal reversion on phytochrome dynamics. This simplified the estimates of PPE to only photoconversions, and therefore *the cellular model*, which accounts for other phytochrome dynamics, could be ignored.

*The three-state model* could still be investigated by simply squaring PPE calculated by *the two-state model* (Mancinelli, 1988). The correlations between PPE and elongation were not greatly changed when using *the three-state* over the *two-state model* (Figures 7B,C compared to Figures 8, 11B,C compared to Figures 11D,E), but the linear models between PPE and lnSER for the estimate of phytochrome homogenously distributed in the whole leaf using *the three-state model* produced nearly parallel lines (more specifically, the slopes were not determined to be significantly different) for the three spectral backgrounds (Figure 8B). This means that a change in PPE is predicted to result in identical changes in elongation for the three spectral backgrounds. These results suggest that *the three-state* model for PPE_whole leaf_ best predicts phytochrome action.

*The three-state model* assuming phytochrome is homogeneously distributed in all leaf tissue provided non-linear relationship between PPE and elongation in the short-term photobleaching study, and while linear responses may be more satisfying, it is possible that the response of stem extension to changes in PPE is non-linear (described above). Overall, it is difficult to conclude anything further regarding *the two-state* vs. *the three-state models*.

Based on the principles of *the cellular model*, an interaction between intensity and percent far-red is expected (i.e., increasing percent far-red should have a more pronounced effect on stem elongation at lower intensities than higher intensities). Although specific effects of intensity have been well documented in the literature (Smith, 1982), the interactions between intensity and percent far-red on the stem length or stem extension rate have been less well documented.

Hitz et al. (2019) applied three FR fractions (1, 7, and 20% FR) to a PPFD of 100 μmol m^–2^ s^–1^ and a PPFD of 400 μmol m^–2^ s^–1^, and saw an increase in stem length both when the percent FR was increased and when the PPFD was decreased, as *the cellular model* would generally predict. However, when the data from Hitz et al. (2019) is considered as a percent increase from the treatments with no added FR, there appears to be no effect of intensity (Supplementary Figure 9). Child and Smith (1987) saw no difference in the relationship between PPE and the change in stem extension rate at intensities between 50 and 150 μmol m^–2^ s^–1^ of white light. Smith (1990) saw only transient changes in stem extension rate when rapidly increasing or decreasing the total intensity while keeping the R:FR ratio constant. Park and Runkle (2018) did not observe an interaction between PPE and intensity on stem length in petunia, geranium or coleus, but they did observe an independent effect of intensity on petunia stem length. These contradictions are difficult to explain because intensity in these studies, unlike our own, dropped below the thresholds described by Sellaro et al. (2019). Our study may not be representative of a traditional cucumber propagation environment because of the high intensity utilized to minimize this thermal reversion. Further studies at various intensities are required to test the robustness of *the cellular model*.

### Blue and Green Responses

Although stem and hypocotyl elongating were primarily explained by changes in PPE, it cannot be ruled out the background spectra would not have significantly different effects on elongation. The high green and high red treatments had roughly the same percentage of blue photons, which make them comparable to each other, but less comparable to the high blue treatment (Table 1), especially on a PPE basis. This is because the blue light receptors, cryptochromes, must be considered. Blue photons decrease stem elongation in cucumbers (Hernández and Kubota, 2016; Snowden et al., 2016). When the data from both the long-term and short-term studies were plotted with PPE (*two-state* or *three-state*) as the independent variable (Figures 7, 8, 11) the background spectral treatments generally increased in elongation in the order of high blue, high green then high red at the same value of PPE. This indicates a role of blue photons (through cryptochrome), and possibly green photons, in shifting the offset of the PPE model. These results are consistent with Park and Runkle (2019).

Research in the last 15 years has indicated that blue and green photons, sensed through the photoreceptor cryptochrome, act in a similarly antagonistic manner as R and FR. For example, green photons were found to reverse the blue induced decrease in hypocotyl elongation (Bouly et al., 2007). This has led to models of cryptochrome action similar to the phytochrome models described above (Procopio et al., 2016). It might be expected that green photons would increase stem elongation similar to FR, but neither Hernández and Kubota (2016) nor Snowden et al. (2016) saw this response in cucumber. Additionally, although Sellaro et al. (2010) demonstrated that a blue/green ratio reliably predicted hypocotyl lengths, their data showed that increasing the flux of green photons, like blue photons, also decreased hypocotyl elongation, but to a lesser extent than the blue photons. It is difficult to determine what caused this green induced decrease in hypocotyl length, but this effect may explain the differences in offsets for the high blue and high green data compared to high red data (Figures 7, 8, 11).

### Future Directions and Potential Improvements

Kusuma and Bugbee (2021) recently outlined six issues with using PPE as a model to predict morphological responses. These included (1) differences in photoconversion coefficients from different studies, (2) multiple phytochromes, (3) thermal reversions, (4) phytochrome intermediates, (5) fluctuations in Ptotal, and (6) spectral distortion by chlorophyll. In this study, photoconversion coefficients derived from measurements of highly pure phytochrome *in vitro* from Lagarias et al. (1987) were used. Our experiments were constructed to primarily obtain effects from phyB and minimize contributions of thermal reversion, but fluctuations in Ptotal and the formation of intermediates were not accounted for. Finally, the results presented here provide evidence that spectral distortion by chlorophyll must be considered in estimating PPE, but several further considerations could improve the robustness of PPE prediction of morphology based on spectral measurements.

As discussed previously, the leaves and cotyledons are likely the primary location of photon perception by phytochrome, but hypocotyls also contribute to photon perception. The planting density in the long-term study was 20 plants per m^2^, which likely led to additional FR enrichment caused by reflection by neighboring plants. Because FR induced auxin signals can move within the plant (Roig-Villanova and Martinez-Garcia, 2016) it is important to determine how FR signals are integrated across different tissues across the plant.

The spectral distortion functions used in this study were derived from Kazarinova-Fukshansky et al. (1985). These distortion functions were calculated from transmission and reflectance measurements using the Kubelka-Munk theory from Kazarinova-Fukshansky et al. (1985), who made their measurements in 7 days old zucchini seedlings grown under 16,000 lx of white light (it is difficult to determine what this is in PPFD, but we estimate that it is about 250–300 μmol m^–2^ s^–1^). Because spectral reflectance and transmittance have roughly the same shape for all plants with chlorophyll, these distortion functions may have relatively universal utility, but environmental conditions contribute to a few key changes in plant internal structure that could decrease the reliability of the presented distortion functions.

#### Potential Shifts in Spectral Distortion Functions

Increasing the FR fraction (decreasing PPE) decreased the leaf chlorophyll concentration (Figure 5D), and there was no effect of percent FR on specific leaf mass, with the exception of a small effect in the high blue treatment (Figure 5E). This means that this change in chlorophyll concentration (μmol per square meter of leaf) was unlikely caused by changes in leaf thickness, but rather was caused by differences in chlorophyll synthesis or retention. Decreasing the concentration of chlorophyll within the leaves is expected to increase the penetration of photons into deeper layers of tissue, increasing the average photon intensity within a leaf. This would result in spectral distortion functions (and thus photoconversion weighting factors) that are intermediate between the epidermis and whole leaf estimates (Figures 4B, 6B,C).

The change in the spectral distortion function with changing chlorophyll concentrations will depend on the distribution of the chlorophyll within the leaves. Nishio et al. (1993) reported that carotenoid and chlorophyll concentrations peaked halfway through a spinach leaf if the plants were grown in sunlight, but peaked at a depth of about 30% through the leaf when grown in the shade. When chlorophyll/carotenoids are concentrated toward the adaxial side of the leaf, photons in the will be attenuated more rapidly, decreasing the average photon flux within the leaf. It seems unlikely that the shade (FR) induced changes in both chlorophyll concentration and chlorophyll distribution will perfectly offset each other, but nonetheless the two effects would antagonistically alter the average SPD within the leaf. If chlorophyll distributions favor the adaxial side under higher FR, this may mean that the FR induced decrease in chlorophyll concentration will minimally affect the average spectral distortion within the leaf.

High photon intensity and blue photons can increase leaf thickness and reorient chloroplasts. Cui et al. (1991) suggested that increased leaf thickness via palisade elongation promoted photon penetration deeper into leaf tissue, although there was little difference in fractional leaf penetration between thick and thin leaves in their study. Chloroplast orientation along the sides of cell walls at high photon intensity induces a sieve effect allowing photon penetration deeper into leaf tissue (Davis et al., 2011; Parry et al., 2014). Again, this results in spectral distortion functions intermediate between the whole leaf and epidermis estimates (Figure 4B).

Developing leaves tend to have lower chlorophyll concentrations than mature leaves. As plants mature and chlorophyll concentrations increase, the average fluxes of blue and red photons within a leaf will decrease. This means that the phytochrome dynamics in older leaves would shift to lower average Pfr concentrations than younger leaves under identical SPD. Younger leaves were more receptive to far-red than older cotyledons in Casal and Smith (1988b). This response is the opposite of what would be expected assuming chlorophyll concentrations were higher in older cotyledon tissue compared to younger leaf tissue. Therefore, younger leaves may be more receptive to photon signals than older leaves. Nonetheless, as these younger leaves develop and chlorophyll concentrations increase, photon penetration into leaves will decrease, shifting the spectral distortion functions from similar to the epidermis estimate to lower than the whole leaf estimate (Figure 4B).

The combined effects of photon quality and quantity on leaf internal structure and chlorophyll concentration/distribution could result in changes in the internal SPD. Modifications to the spectral distortion functions to account for these changes could improve the model. Additional research is warranted.

### A Simpler Intuitive Metric: The FR Fraction

Phytochrome and cryptochrome, when activated, interact with some of the same transcription factors (de Wit et al., 2016). The chromophore at the center of the photoreceptor cryptochrome is a flavin adenine dinucleotide (FAD) molecule, a coenzyme associated with numerous proteins. FAD absorbs photons in the UV-A and blue regions of the spectrum. FAD absorbance drops substantially around 500 nm (Banerjee et al., 2007; Procopio et al., 2016). The inactive form of phytochrome absorbs across the entire biologically active spectrum (300–800 nm), but is primarily activated by red photons. Chlorophyll-induced spectral distortions may mean that phytochrome is also significantly activated by (longer wavelength) green photons (Figure 6C). Therefore, blue, green and red photons may push back against FR photons to affect morphology. Percent far-red (FR fraction) was shown to be an excellent predictor of lnSER in the long-term study (*r*^2^ = 0.89, Figure 5A), although the expected blue (and possibly green) offsets are not present. Percent far-red did not appear to be a good predictor of morphology in seedlings (Figure 10).

Due to the issues with PPE outlines above, Kusuma and Bugbee (2021) suggested that environmental signals may be more reliable than photo-molecular models, like PPE. Environmental pressure drives evolution, and thus genetically regulated molecular machinery could be expected to conform to the incoming signals (in so much as it provides a survival advantage). The R:FR ratio is often used as a metric to describe the degree of shade, but percent far-red may be a better ratio because it integrates the action of multiple photoreceptors that co-evolved to detect the extent of shade. Although our improvements to the PPE model indicate some important mechanistic aspects of photon perception within a leaf, the FR fraction is a simple intuitive metric that may be widely applicable across many conditions.

## Summary

Phytochrome photoequilibrium is generally estimated from the SPD above the leaf, which does not account for the spectral distortion caused by absorbance and scattering within a leaf, and is thus an inadequate metric for estimating phytochrome induced morphology. Estimates of PPE for phytochrome that is homogeneously distributed throughout the whole leaf accounted for spectral distortions and was a better predictor of morphological responses. The distortion functions used here were from a different species than species investigated and yet improved predictions. We thus believe the distortion functions used here have universal utility. We provide both the distortion functions and photoconversion weighting factors in Supplementary Data.

Percent far-red is an intuitive environmental metric that accounts for photon effects from 400 to 750 nm on stem elongation rate, possibly because it accounts for cryptochrome and phytochrome action. This is an empirical metric but it appears to have excellent predictive power.

The use of LEDs in controlled environments allows an unprecedented opportunity to manipulate plant growth. FR LEDs have a high efficacy and may thus contribute to these manipulations, but the phytochrome mediated responses to FR must be better understood to utilize their potential.

## Data Availability Statement

All datasets presented in this study are included in the article/Supplementary Material.

## Author Contributions

PK and BB contributed to the design of the study, analysis of data, and writing of the manuscript. Both authors contributed to the article and approved the submitted version.

## Conflict of Interest

The authors declare that the research was conducted in the absence of any commercial or financial relationships that could be construed as a potential conflict of interest.

## Footnotes

1 http://getdata-graph-digitizer.com

## References

[B1] Aukerman M. J. Hirschfeld M. Wester L. Weaver M. Clack T. Amasino R. M. (1997). A deletion in the PHYD gene of the Arabidopsis Wassilewskija ecotype defines a role for phytochrome D in red/far-red light sensing. *Plant Cell* 9 1317–1326. 10.1105/tpc.9.8.1317 9286109 PMC157000

[B2] Banerjee R. Schleicher E. Meier S. Muñoz Viana R. Pokorny R. Ahmad M. (2007). The signaling state of Arabidopsis cryptochrome 2 contains flavin semiquinone. *J. Biol. Chem.* 282 14916–14922. 10.1074/jbc.M700616200 17355959

[B3] Barnes C. Bugbee B. (1992). Morphological responses of wheat to blue light. *J. Plant Physiol.* 139 339–342. 10.1016/S0176-1617(11)80347-0 11537086

[B4] Björkman T. (1999). Dose and timing of brushing to control excessive hypocotyl elongation in cucumber transplants. *HortTechnology* 9 224–226. 10.21273/HORTTECH.9.2.224

[B5] Black M. Shuttleworth J. E. (1974). The role of the cotyledons in the photocontrol of hypocotyl extension in Cucumis sativus L. *Planta* 117 57–66. 10.1007/BF00388678 24458299

[B6] Borthwick H. A. Hendricks S. B. Parker M. W. Toole E. H. Toole V. K. (1952). A reversible photoreaction controlling seed germination. *Proc. Natl. Acad. Sci. U.S.A.* 38 662–666. 10.1073/pnas.38.8.662 16589159 PMC1063632

[B7] Bouly J. P. Schleicher E. Dionisio-Sese M. Vandenbussche F. Van Der Straeten D. Bakrim N. (2007). Cryptochrome bluelight photoreceptors are activated through interconversion of flavinredox states. *J. Biol. Chem.* 282 9383–9391. 10.1074/jbc.M609842200 17237227

[B8] Brockmann J. Rieble S. Kazarinova-Fukshansky N. Seyfried M. Schäfer E. (1987). Phytochrome behaves as a dimer in vivo. *Plant Cell Environ.* 10 105–111. 10.1111/1365-3040.ep11602037

[B9] Butler W. L. Hendricks S. B. Siegelman H. W. (1964). Action spectra of phytochrome in vitro. *Photochem. Photobiol.* 3 521–528. 10.1111/j.1751-1097.1964.tb08171.x

[B10] Casal J. J. (1995). Coupling of phytochrome B to the control of hypocotyl growth in Arabidopsis. *Planta* 196 23–29. 10.1007/BF00193213 7767236

[B11] Casal J. J. Smith H. (1988a). Persistent effects of changes in phytochrome status on internode growth in light-grown mustard: occurrence, kinetics and locus of perception. *Planta* 175 214–220. 10.1007/BF00392430 24221715

[B12] Casal J. J. Smith H. (1988b). The loci of perception for phytochrome control of internode growth in light-grown mustard: promotion by low phytochrome photoequilibria in the internode is enhanced by blue light perceived by the leaves. *Planta* 176 277–282. 10.1007/BF00392456 24220784

[B13] Child R. Smith H. (1987). Phytochrome action in light-grown mustard: kinetics, fluence-rate compensation and ecological significance. *Planta* 172 219–229. 10.1007/BF00394591 24225874

[B14] Cope K. R. Bugbee B. (2013). Spectral effects of three types of white light-emitting diodes on plant growth and development: absolute versus relative amounts of blue light. *HortScience* 48 504–509. 10.21273/HORTSCI.48.4.504

[B15] Cui M. Vogelmann T. C. Smith W. K. (1991). Chlorophyll and light gradients in sun and shade leaves of Spinacia oleracea. *Plant Cell Environ.* 14 493–500. 10.1111/j.1365-3040.1991.tb01519.x

[B16] Davis P. A. Caylor S. Whippo C. W. Hangarter R. P. (2011). Changes in leaf optical properties associated with light-dependent chloroplast movement. *Plant Cell Environ.* 34 2047–2059. 10.1111/j.1365-3040.2011.02402.x 21819411

[B17] de Lucas M. Prat S. (2014). PIFs get BRright: PHYTOCHROME INTERACTING FACTORs as integrators of light and hormonal signals. *New Phytol.* 202 1126–1141. 10.1111/nph.12725 24571056

[B18] de Wit M. Keuskamp D. H. Bongers F. J. Hornitschek P. Gommers C. M. Reinen E. (2016). Integration of phytochrome and cryptochrome signals determines plant growth during competition for light. *Curr. Biol.* 26 3320–3326. 10.1016/j.cub.2016.10.031 27889265

[B19] Devlin P. F. Patel S. R. Whitelam G. C. (1998). Phytochrome E influences internode elongation and flowering time in Arabidopsis. *Plant Cell* 10 1479–1487. 10.1105/tpc.10.9.1479 9724694 PMC144080

[B20] Devlin P. F. Robson P. R. Patel S. R. Goosey L. Sharrock R. A. Whitelam G. C. (1999). Phytochrome D acts in the shade-avoidance syndrome in Arabidopsis by controlling elongation growth and flowering time. *Plant Physiol.* 119 909–916. 10.1104/pp.119.3.909 10069829 PMC32105

[B21] Dougher T. A. Bugbee B. (2001a). Differences in the Response of Wheat, Soybean and Lettuce to Reduced Blue Radiation. Photochem. *Photobiol.* 73 199–207. 10.1562/0031-865520010730199DITROW2.0.CO2 11272735

[B22] Dougher T. A. Bugbee B. (2001b). Evidence for yellow light suppression of lettuce growth. *Photochem. Photobiol.* 73 208–212. 10.1562/0031-865520010730208EFYLSO2.0.CO2 11272736

[B23] Eichenberg K. Bäurle I. Paulo N. Sharrock R. A. Rüdiger W. Schäfer E. (2000). Arabidopsis phytochromes C and E have different spectral characteristics from those of phytochromes A and B. *FEBS Lett.* 470 107–112. 10.1016/S0014-5793(00)01301-6 10734217

[B24] Endo M. Nakamura S. Araki T. Mochizuki N. Nagatani A. (2005). Phytochrome B in the mesophyll delays flowering by suppressing FLOWERING LOCUS T expression in Arabidopsis vascular bundles. *Plant Cell* 17 1941–1952. 10.1105/tpc.105.032342 15965119 PMC1167543

[B25] Evans J. R. (1995). Carbon fixation profiles do reflect light absorption profiles in leaves. *Aust. J. Plant Physiol.* 22 865–873. 10.1071/PP9950865

[B26] Fisher P. R. Heins R. D. Lieth J. H. (1996). Quantifying the relationship between phases of stem elongation and flower initiation in poinsettia. *J. Am. Soc. Hort. Sci.* 121 686–693. 10.21273/JASHS.121.4.686

[B27] Franklin K. A. Davis S. J. Stoddart M. W. Vierstra R. D. Whitelam G. C. (2003). Mutant analyses define multiple roles for phytochrome C in Arabidopsis photomorphogenesis. *Plant Cell* 15 1981–1989. 10.1105/tpc.015164 12953105 PMC181325

[B28] Franklin K. A. Quail P. H. (2010). Phytochrome functions in Arabidopsis development. *J. Expt. Bot.* 61 11–24. 10.1093/jxb/erp304 19815685 PMC2800801

[B29] García-Martínez J. L. Keith B. Bonner B. A. Stafford A. E. Rappaport L. (1987). Phytochrome regulation of the response to exogenous gibberellins by epicotyls of Vigna sinensis. *Plant Physiol.* 85 212–216. 10.1104/pp.85.1.212 16665660 PMC1054231

[B30] Gardner G. Graceffo M. A. (1982). The use of a computerized spectroradiometer to predict phytochrome photoequilibria under polychromatic irradiation. *Photochem. Photobiol.* 36 349–354. 10.1111/j.1751-1097.1982.tb04385.x

[B31] Hartmann K. M. (1966). A general hypothesis to interpret ‘high energy phenomena’ of photomorphogenesis on the basis of phytochrome. *Photochem. Photobiol.* 5 349–365. 10.1111/j.1751-1097.1966.tb05937.x

[B32] Hernández R. Kubota C. (2016). Physiological responses of cucumber seedlings under different blue and red photon flux ratios using LEDs. *Environ. Exp. Bot.* 121 66–74. 10.1016/j.envexpbot.2015.04.001

[B33] Hitz T. Hartung J. Graeff-Hönninger S. Munz S. (2019). Morphological response of soybean (*Glycine max* (L.) Merr.) cultivars to light intensity and red to far-red ratio. *Agronomy* 9 428. 10.3390/agronomy9080428

[B34] Holmes M. G. Fukshansky L. (1979). Phytochrome photoequilibria in green leaves under polychromatic radiation: a theoretical approach. *Plant Cell Environ.* 2 59–65. 10.1111/j.1365-3040.1979.tb00774.x

[B35] Holmes M. G. Wagner E. (1981). Phytochrome control of hypocotyl extension in light-grown Chenopodium rubrum. *Physiologia Plantarum* 53 233–238. 10.1111/j.1399-3054.1981.tb04492.x

[B36] Hughes J. (2013). Phytochrome cytoplasmic signaling. *Annu. Rev. Plant Biol.* 64 377–402. 10.1146/annurev-arplant-050312-120045 23506333

[B37] Jones A. M. Quail P. H. (1986). Quaternary structure of 124-kilodalton phytochrome from *Avena sativa* L. *Biochemistry* 25 2987–2995. 10.1021/bi00358a038

[B38] Jose A. M. Schäfer E. (1978). Distorted phytochrome action spectra in green plants. *Planta* 138 25–28. 10.1007/BF00392909 24413936

[B39] Kasperbauer M. J. Borthwick H. A. Hendricks S. B. (1963). Inhibition of flowering of Chenopodium rubrum by prolonged far-red radiation. *Bot. Gaz.* 124 444–451. 10.1086/336234

[B40] Kazarinova-Fukshansky N. Seyfried M. Schäfer E. (1985). Distortion of action spectra in photomorphogenesis by light gradients within the plant tissue. *Photochem. Photobiol.* 41 689–702. 10.1111/j.1751-1097.1985.tb03624.x

[B41] Kelly J. M. Lagarias J. C. (1985). Photochemistry of 124-kilodalton avena phytochrome under constant illumination in vitro. *Biochemistry* 24 6003–6010. 10.1021/bi00342a047

[B42] Kim J. Song K. Park E. Kim K. Bae G. Choi G. (2016). Epidermal phytochrome B inhibits hypocotyl negative gravitropism non-cell-autonomously. *Plant Cell* 28 2770–2785. 10.1105/tpc.16.00487 27758895 PMC5155346

[B43] Klose C. Venezia F. Hussong A. Kircher S. Schäfer E. Fleck C. (2015). Systematic analysis of how phytochrome B dimerization determines its specificity. *Nat. Plants* 1 15090. 10.1038/nplants.2015.90 27250256

[B44] Kusuma P. Bugbee B. (2021). Far-red Fraction: an improved metric for characterizing phytochrome effects on morphology. *J. Am. Soc. Hort. Sci.* 146 3–13. 10.21273/JASHS05002-20

[B45] Kutschera U. Niklas K. J. (2007). The epidermal-growth-control theory of stem elongation: an old and a new perspective. *J. Plant Physiol.* 164 1395–1409. 10.1016/j.jplph.2007.08.002 17905474

[B46] Lagarias J. C. Kelly J. M. Cyr K. L. Smith W. O. Jr. (1987). Comparative photochemical analysis of highly purified 124 kilodalton oat and rye phytochromes in vitro. *Photochem. Photobiol.* 46 5–13. 10.1111/j.1751-1097.1987.tb04729.x

[B47] Legris M. Ince Y. Ç Fankhauser C. (2019). Molecular mechanisms underlying phytochrome-controlled morphogenesis in plants. *Nat. Commun.* 10:5219. 10.1038/s41467-019-13045-0 31745087 PMC6864062

[B48] Mancinelli A. L. (1986). Comparison of spectral properties of phytochromes from different preparations. *Plant physiol.* 82 956–961. 10.1104/pp.82.4.956 16665173 PMC1056240

[B49] Mancinelli A. L. (1988). Some thoughts about the use of predicted values of the state of phytochrome in plant photomorphogenesis research. *Plant Cell Environ.* 11 429–439. 10.1111/j.1365-3040.1988.tb01780.x

[B50] Mancinelli A. L. (1994). “The physiology of phytochrome action,” in *Photomorphogenesis in Plants*, eds Kendrick R. E. Kronenberg G. H. M. (Netherlands: Springer), 211–269. 10.1007/978-94-011-1884-2_10

[B51] Mazzella M. A. Casal J. J. (2001). Interactive signalling by phytochromes and cryptochromes generates de-etiolation homeostasis in Arabidopsis thaliana. *Plant Cell Environ.* 24 155–161. 10.1111/j.1365-3040.2001.00653.x

[B52] Morgan D. Smith H. (1978). The relationship between phytochrome-photoequilibrium and development in light grown *Chenopodium album* L. *Planta* 142 187–193. 10.1007/BF00388211 24408101

[B53] Morgan D. C. Smith H. (1976). Linear relationship between phytochrome photo-equilibrium and growth in plants under simulated natural radiation. *Nature* 262 210–212. 10.1038/262210a0

[B54] Morgan D. C. Smith H. (1979). A systematic relationship between phytochrome controlled development and species habitat. *Planta* 145 253–258. 10.1007/BF00454449 24317731

[B55] Nishio J. N. Sun J. D. Vogelmann T. C. (1993). Carbon fixation gradients across spinach leaves do not follow internal light gradients. *Plant Cell* 5 953–961. 10.1105/tpc.5.8.953 12271092 PMC160330

[B56] Park Y. Runkle E. S. (2017). Far-red radiation promotes growth of seedling by increasing leaf expansion and whole-plant net assimilation. *Environ. Exp. Bot.* 136 41–49. 10.1016/j.envexpbot.2016.12.013

[B57] Park Y. Runkle E. S. (2018). Far-red radiation and photosynthetic photon flux density independently regulate seedling growth but interactively regulate flowering. *Environ. Exp. Bot.* 155 206–216. 10.1016/j.envexpbot.2018.06.033

[B58] Park Y. Runkle E. S. (2019). Blue radiation attenuates the effects of the red to far-red ratio on extension growth but not on flowering. *Environ. Exp. Bot.* 168:103871. 10.1016/j.envexpbot.2019.103871

[B59] Parry C. Blonquist J. M. Bugbee B. (2014). In situ measurement of leaf chlorophyll concentration: analysis of the optical/absolute relationship. *Plant Cell Environ.* 37 2508–2520. 10.1111/pce.12324 24635697

[B60] Procko C. Crenshaw C. M. Ljung K. Noel J. P. Chory J. (2014). Cotyledon-generated auxin is required for shade-induced hypocotyl growth in Brassica rapa. *Plant Physiol.* 165 1285–1301. 10.1104/pp.114.241844 24891610 PMC4081337

[B61] Procopio M. Link J. Engle D. Witczak J. Ritz T. Ahmad M. (2016). Kinetic modeling of the Arabidopsis cryptochrome photocycle: FADH accumulation correlates with biological activity. *Front. Plant Sci.* 7:888. 10.3389/fpls.2016.00888 27446119 PMC4924484

[B62] Rausenberger J. Hussong A. Kircher S. Kirchenbauer D. Timmer J. Nagy F. (2010). An integrative model for phytochrome B mediated photomorphogenesis: from protein dynamics to physiology. *PLoS One* 5:e10721. 10.1371/annotation/4563eaf4-e45b-4d9e-ab06-5f1794bf11e3 PMC2873432 20502669

[B63] Rockwell N. C. Su Y. Lagarias J. C. (2006). Phytochrome structure and signaling mechnaisms. *Annu. Rev. Plant Biol.* 57 837–858. 10.1146/annurev.arplant.56.032604.144208 16669784 PMC2664748

[B64] Roig-Villanova I. Martinez-Garcia J. F. (2016). Plant responses to vegetation proximity: a whole life avoiding shade. *Front. Plant Sci.* 7:236. 10.3389/fpls.2016.00236 26973679 PMC4770057

[B65] Ruddat A. Schmidt P. Gatz C. Braslavsky S. E. Gärtner W. Schaffner K. (1997). Recombinant type A and B phytochromes from potato. Transient absorption spectroscopy. *Biochemistry* 36 103–111. 10.1021/bi962012w 8993323

[B66] Sage L. C. (1992). *Pigment of the Imagination.* Boston: Academic Press, 562.

[B67] Sager J. C. Smith W. O. Edwards J. L. Cyr K. L. (1988). Photosynthetic efficiency and phytochrome photoequilibria determination using spectral data. *Trans. ASAE* 31 1882–1889. 10.13031/2013.30952

[B68] Savaldi-Goldstein S. Peto C. Chory J. (2007). The epidermis both drives and restricts plant shoot growth. *Nature* 446 199–202. 10.1038/nature05618 17344852

[B69] Sellaro R. Crepy M. Trupkin S. A. Karayekov E. Buchovsky A. S. Rossi C. (2010). Cryptochrome as a sensor of the blue/green ratio of natural radiation in Arabidopsis. *Plant Physiol.* 154 401–409. 10.1104/pp.110.160820 20668058 PMC2938137

[B70] Sellaro R. Smith R. W. Legris M. Fleck C. Casal J. J. (2019). Phytochrome B dynamics departs from photoequilibrium in the field. *Plant Cell Environ.* 42 606–617. 10.1111/pce.13445 30216475

[B71] Seyfried M. Fukshansky L. (1983). Light gradients in plant tissue. *Appl. Optics* 22 1402–1408. 10.1364/AO.22.001402 18195976

[B72] Sharrock R. A. Clack T. (2002). Patterns of expression and normalized levels of the five Arabidopsis phytochromes. *Plant Physiol.* 130 442–456. 10.1104/pp.005389 12226523 PMC166576

[B73] Smith H. (1973). “Light quality and germination: ecological implications,” in *Seed ecology*, ed. Heydecker W. (Butterworths), 219–231.

[B74] Smith H. (1975). “The photomorphogenic response systems and their photoreceptors,” in *Phytochrome and Photomorphogenesis*, ed. Smith H. (London: McGraw-Hill), 22–53.

[B75] Smith H. (1982). Light quality, photoperception, and plant strategy. *Annu. Rev. Plant Physiol.* 33 481–518. 10.1146/annurev.pp.33.060182.002405

[B76] Smith H. (1990). Phytochrome action at high photon fluence rates: rapid extension rate responses of light-grown mustard to variations in fluence rate and red: far-red ratio. *Photochem. Photobiol.* 52 131–142. 10.1111/j.1751-1097.1990.tb01766.x

[B77] Smith R. W. Fleck C. (2019). “Basic phytochrome B calculations,” in *Phytochromes: Methods and Protocols*, ed. Hiltbrunner A. (New York, NY: Humana), 121–133. 10.1007/978-1-4939-9612-4_9 31317407

[B78] Snowden C. M. Cope K. R. Bugbee B. (2016). Sensitivity of seven diverse species to blue and green light: integrations with photon flux. *PLoS One* 11:e0163121. 10.1371/journal.pone.0163121 27706176 PMC5051895

[B79] Somers D. E. Quail P. H. (1995). Temporal and spatial expression patterns of PHYA and PHYB genes in Arabidopsis. *Plant J.* 7 413–427. 10.1046/j.1365-313X.1995.7030413.x 7757114

[B80] Tanaka S. I. Nakamura S. Mochizuki N. Nagatani A. (2002). Phytochrome in cotyledons regulates the expression of genes in the hypocotyl through auxin-dependent and-independent pathways. *Plant Cell Physiol.* 43 1171–1181. 10.1093/pcp/pcf133 12407197

[B81] USU Crop Physiology Laboratory (2020). “Utah monocot/dicot solution,” in *Nutrients. Paper 2.* Available online at: https://digitalcommons.usu.edu/cgi/viewcontent.cgi?article=1001&context=cpl_nutrients (accessed December 10, 2020).

[B82] Vogelmann T. C. (1994). “Light within the plant,” in *Photomorphogenesis in Plants*, eds Kendrick R. E. Kronenberg G. H. M. (Netherlands: Springer), 491–535. 10.1007/978-94-011-1884-2_18

[B83] Whitelam G. C. Johnson E. Peng J. Carol P. Anderson M. L. Cowl J. S. (1993). Phytochrome a null mutants of Arabidopsis display a wild-type phenotype in white light. *Plant Cell* 5 757–768. 10.1105/tpc.5.7.757 8364355 PMC160314

[B84] Zhen S. Bugbee B. (2020). Far-red photons have equivalent efficiency to traditional photosynthetic photons: Implications for redefining photosynthetically active radiation. *Plant Cell Environ.* 43 1259–1272. 10.1111/pce.13730 31990071

[B85] Zhen S. van Iersel M. W. (2017). Far-red light is needed for efficient photochemistry and photosynthesis. *J. Plant Physiol.* 209 115–122. 10.1016/j.jplph.2016.12.004 28039776

[B86] Zhen S. Y. Haidekker M. van Iersel M. W. (2018). Far-red light enhances photochemical eficiency in a wavelength-dependent manner. *Physiol. Plant* 167 21–33. 10.1111/ppl.12834 30203475

