# Supplementary material for: Improving the Predictive Value of Phytochrome Photoequilibrium: Consideration of Spectral Distortion Within a Leaf
Source: Front Plant Sci. 2021 May 24;12:596943. doi: 10.3389/fpls.2021.596943 (PMC8181145; doi:10.3389/fpls.2021.596943)

Table S1

Percent far-red in each treatment. There were 7 trials in time, each with a higher and lower dose of FR.

	Trial #	FR Dose	HIGH BLUE	HIGH GREEN	HIGH RED
% FAR-RED $\frac{\sum 701 - 750\,nm}{\sum 400 - 750\,nm}$	Trial 1	LOW	1.5	3.4	1.3
		HIGH	9.1	12	8.6
	Trial 2	LOW	1.5	3.2	1.8
		HIGH	9.2	9.0	9.2
	Trial 3	LOW	1.6	5.5	1.3
		HIGH	25	24	23
	Trial 4	LOW	1.6	6.3	1.4
		HIGH	25	25	25
	Trial 5	LOW	13	13	13
		HIGH	19	20	18
	Trial 6	LOW	13	13	12
		HIGH	20	20	20
	Trial 7	LOW	18	17	16
		HIGH	41	44	39

Fig. S1

Effect of percent far-red on stem length and leaf area in tomato

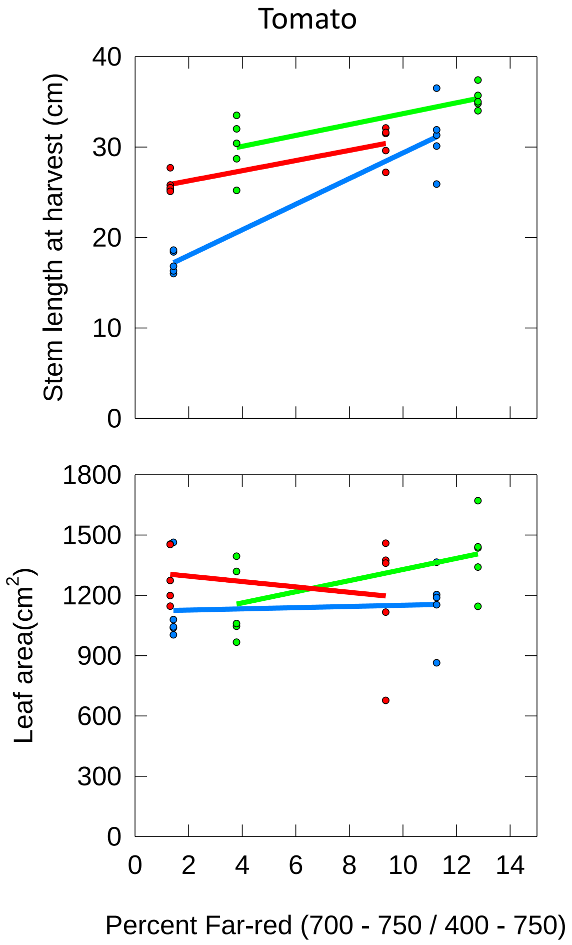

Fig. S2

Photo of the chambers used in the study. FR LEDs were placed on either side of the background spectrum for one half of the chamber. These were angled inward to avoid contamination with the other side.

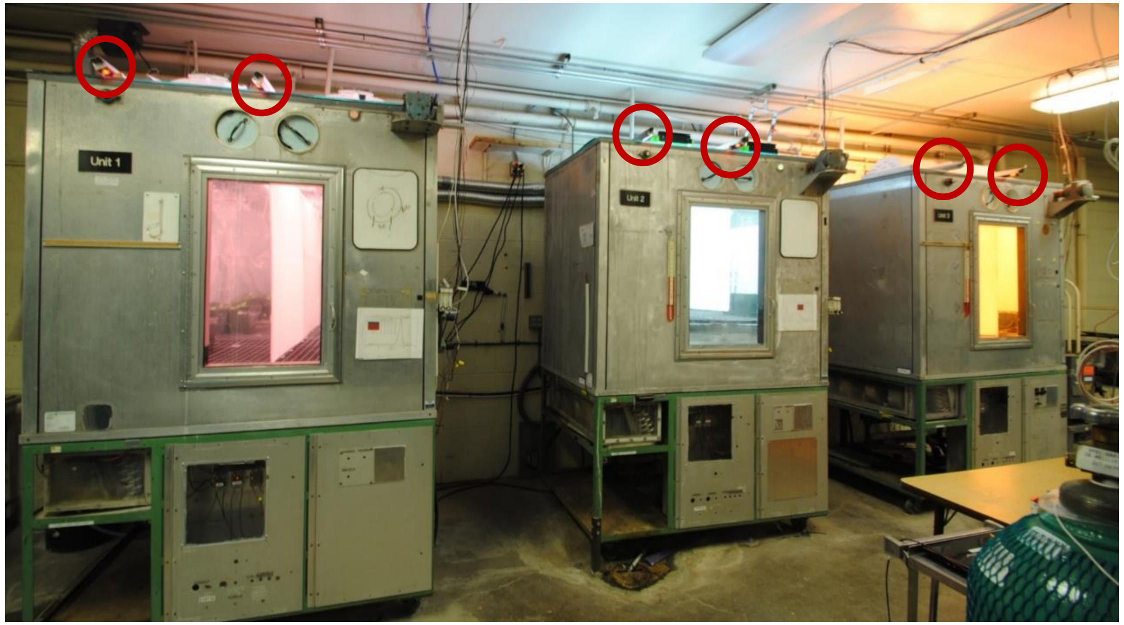

Fig. S3

Reflectance of the black felt

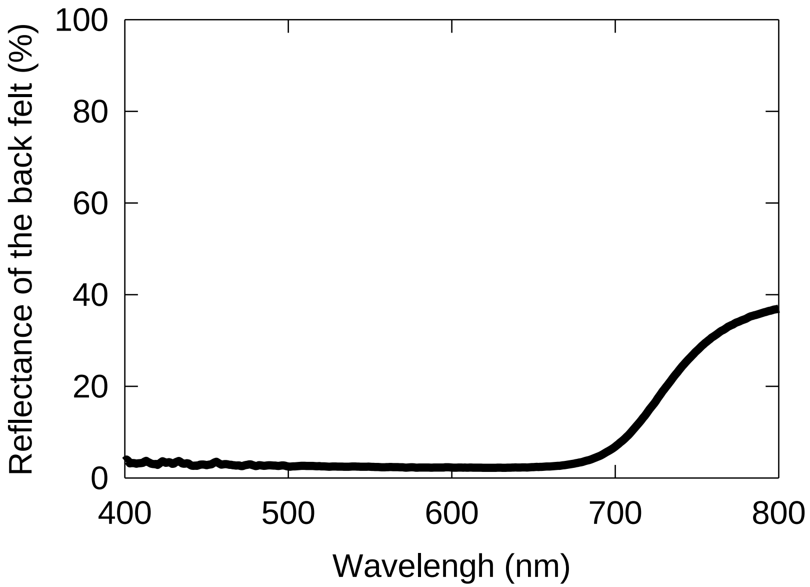

Fig. S4

Spectrum of the pre-treatment for the short-term photobleached seedling study

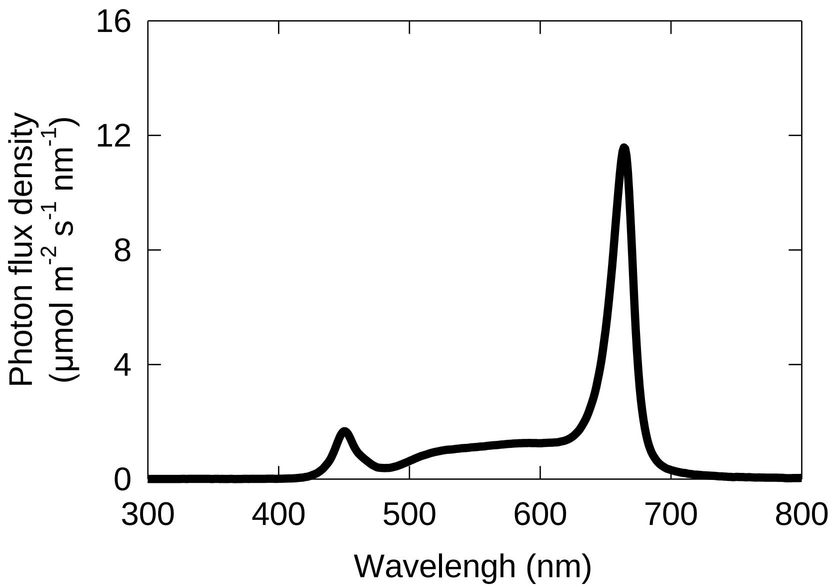

Fig. S5

Average spectral photon distribution in each FR dose treatment. Blue , green and red lines represent the high blue, green and red background treatments, respectively.

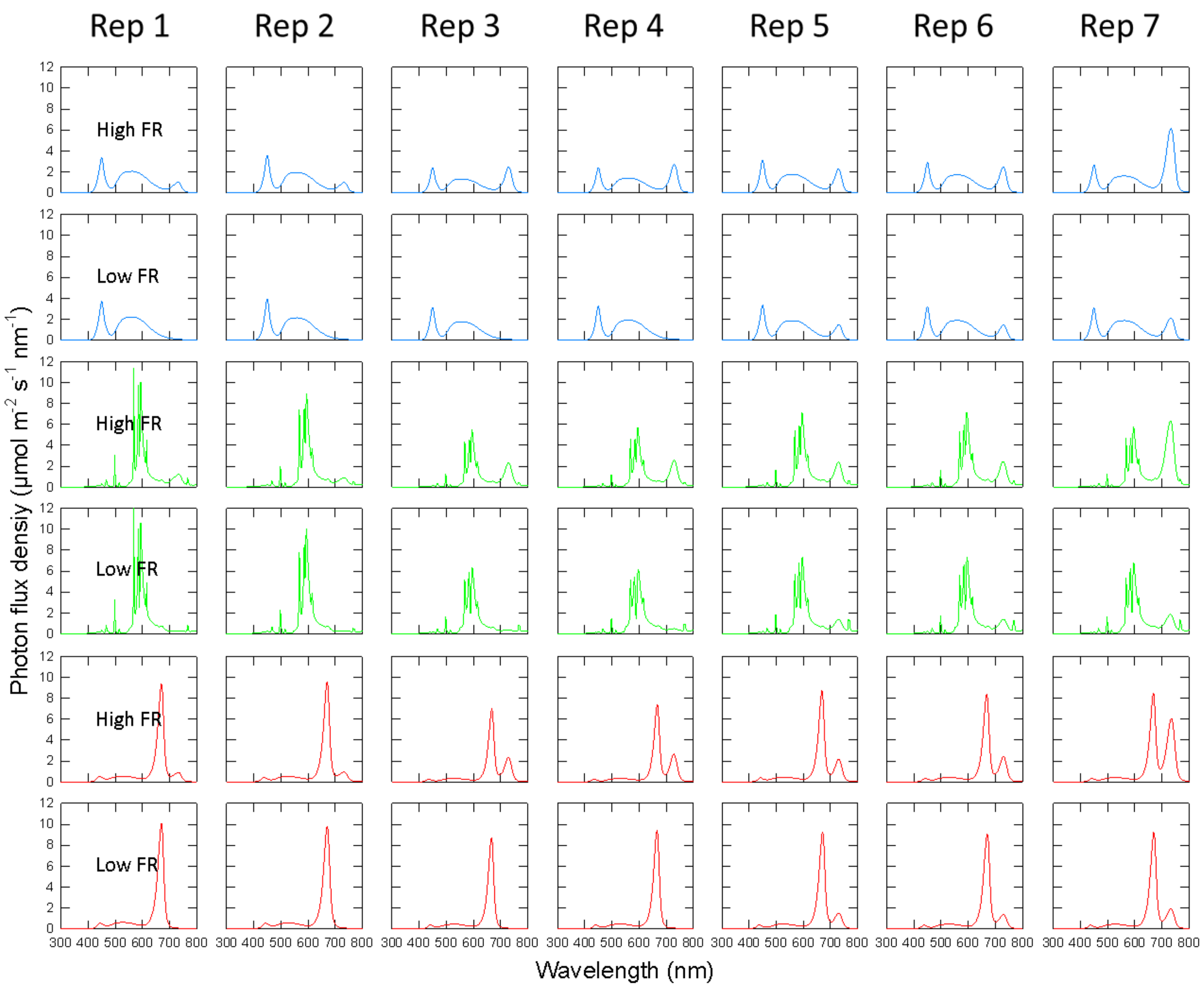

Fig. S6

Spectral treatments from the short-term photobleaching study. These experiments used the same spectral backgrounds as the long-term study. These studies only contained three levels of FR. Measurements are averages from four replicate studies.

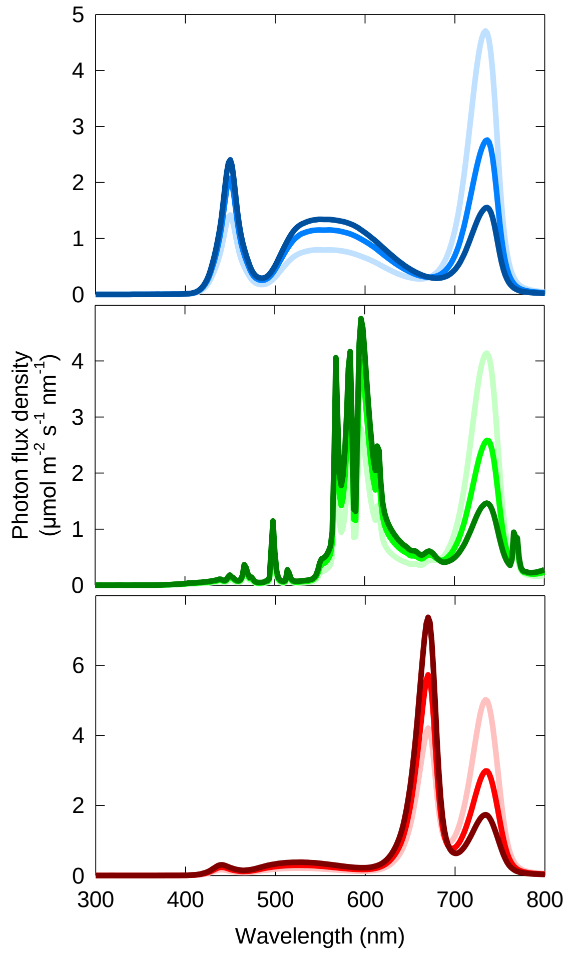

Fig. S7

Photoconversion weighting factors for phytochrome conversion in etiolated tissue. Weighting factors are for (a) phytochrome in the epidermal tissue or (b) phytochrome homogeneously distributed through all cotyledon tissue.

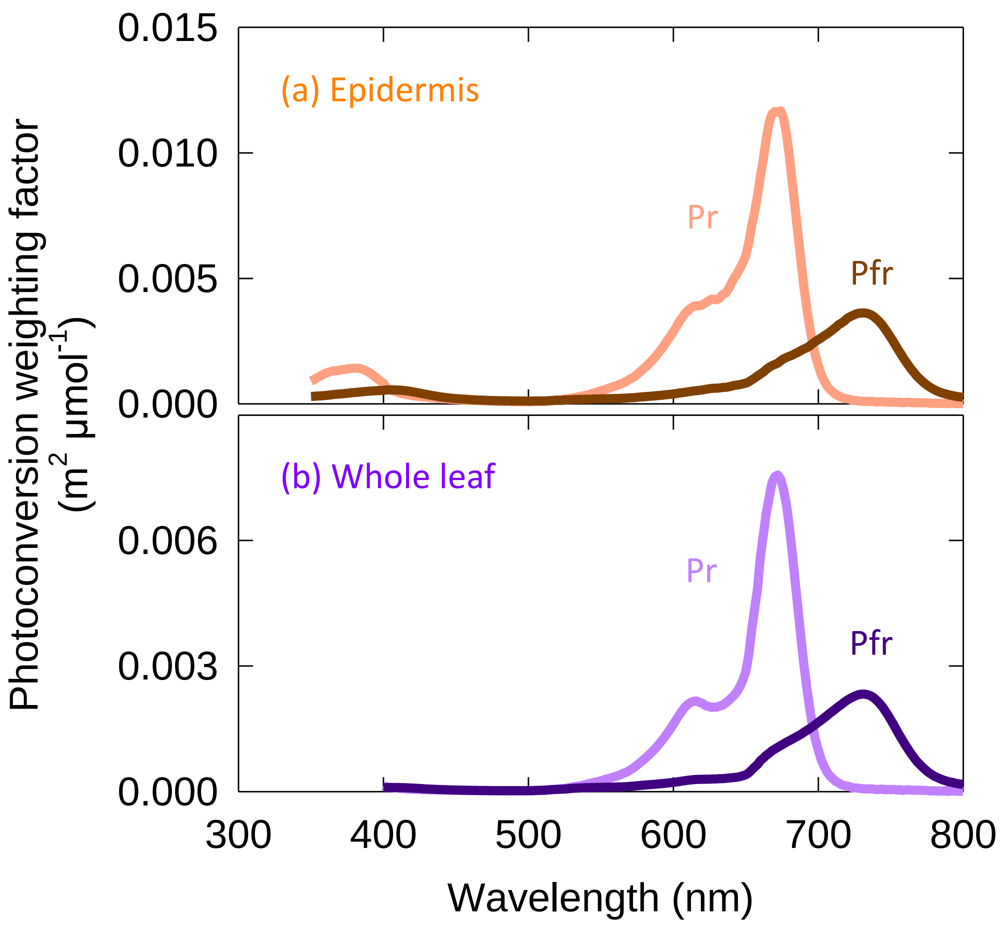

Fig. S8

Relationship between lnSER in the long term study and PPE estimated using the transmission spectrum as a distortion function. The relationship is non-linear.

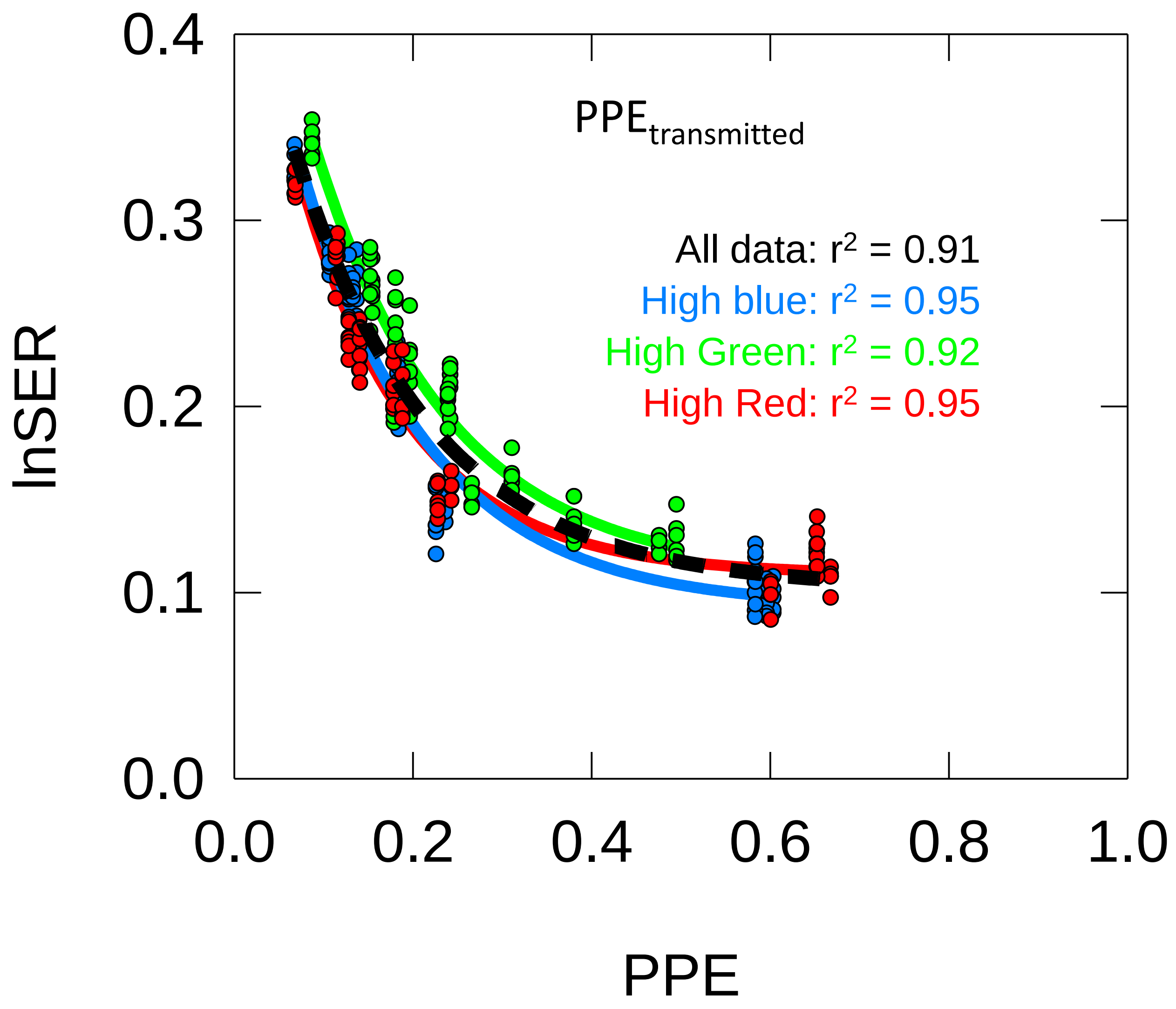

Fig. S9

Data from Hitz et al. (2019) represented in graphs that use percent FR. Green data comes from the cv. Lissabon, red data comes from the cv. Merlin, and blue data comes from the cv. Sultana. Darker lines are were grown under a PPFD of 100 µmol m^{-2} s^{-1} and the dark lines were grown under 400 µmol m^{-2} s^{-1}. (a) The data graphed with absolute tem length. (b) the data re-graphed as percent increase compared to the treatment with no added FR.

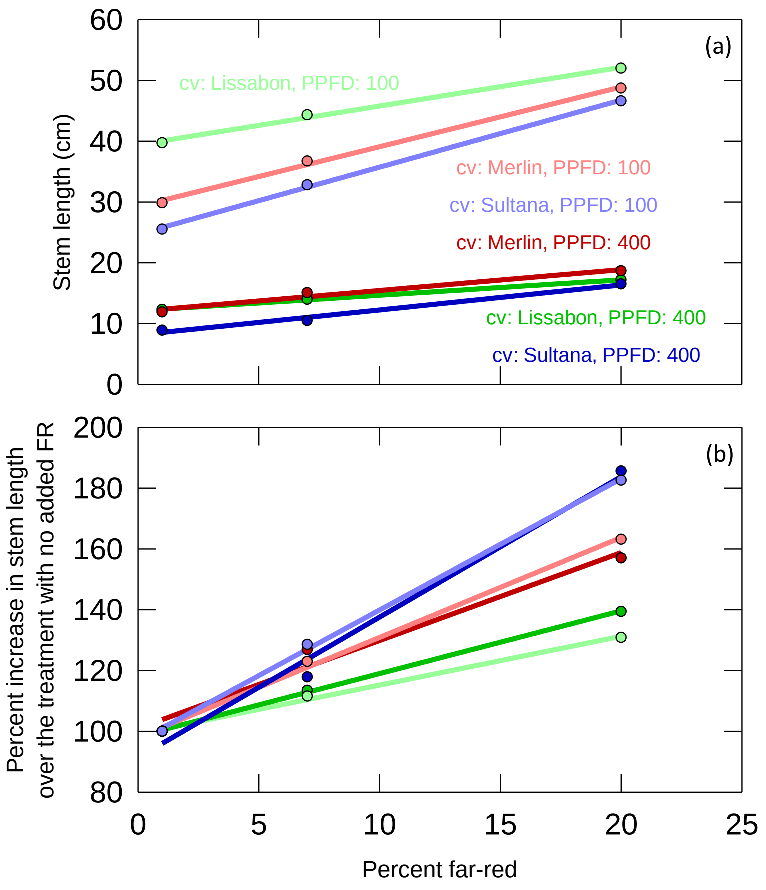

Supplement: Supplementary file 1 [file Data_Sheet_1.PDF]
